# Trends in medical debt in the United States by imputed borrower race and ethnicity, 2016-2022

**DOI:** 10.1093/haschl/qxag091

**Published:** 2026-05-22

**Authors:** Alexander C Adia, Jenny S Guadamuz, Charleen Hsuan, Hector P Rodriguez

**Affiliations:** Division of Health Policy and Management, School of Public Health, University of California, Berkeley, CA 94704, United States; Division of Health Policy and Management, School of Public Health, University of California, Berkeley, CA 94704, United States; Department of Health Policy & Administration, Pennsylvania State University, University Park, PA 16802, United States; Division of Health Policy and Management, School of Public Health, University of California, Berkeley, CA 94704, United States

**Keywords:** medical debt, race and ethnicity, disparities, Medicaid expansion, health insurance

## Abstract

**Introduction:**

Medical debt is a major financial consequence of healthcare in the United States, but there is limited evidence about whether medical debt in collections varies across racial and ethnic groups.

**Methods:**

We analyzed credit report data from 2016 to 2022 for 23,093,888 adults to study medical debt in collections accrued within the past year (flow) and total outstanding medical debt in collections (stock).

**Results:**

Black borrowers had the highest levels of annual flow of medical debt, with 11.2% of borrowers facing any annual flow of debt in 2022 (mean conditional on any flow: $1524). Similar trends existed for stock, with 24% of borrowers having any stock in 2022 (mean conditional on any stock: $2074). Hispanic and White borrowers had similar medical debt, and Asian and Pacific Islander borrowers had the least debt. Gaps between racial and ethnic groups were smaller in states that expanded Medicaid compared to those that did not, especially on the flow of new medical debt.

**Discussion:**

Identifying evidence-based policies and interventions to reduce medical debt differences by borrower race and ethnicity should be a high policy priority.

## Introduction

Increasing healthcare costs present financial burdens to many Americans. The Consumer Financial Protection Bureau estimates that 1 in 5 US households face medical debts.^1^ Financial strain from medical debt can increase risk of food or housing insecurity.^2^ Medical debt is frequently cited as a key contributor to personal bankruptcy, even after the Affordable Care Act.^3^ In some cases, patients have reported being denied care due to outstanding medical debt.^4^ Patients can have their debts sent for collections and added to patients’ credit reports, sold to third parties, and pursued in lawsuits where patients may be subject to liens and wage garnishment.^5^

Medical debt has major implications for personal health and finances,^2,6^ drawing bipartisan support for policy action and reform.^7^ Several local and state governments have purchased and forgiven medical debt owed by their constituents.^8-11^ Recent policies by the major credit bureaus include voluntarily excluding medical debt under $500, paid medical debts, and debts under a year old from credit reports.^12^

Medical debt will continue to burden Americans and may exacerbate health disparities if unaddressed, making the need to understand differences in debt by race and ethnicity critical for informing policy.^13^ Court records in Wisconsin^14^ and Missouri^15^ indicate that lawsuits to recover unpaid medical debt are disproportionately targeted at Black patients. Prior studies suggest substantial disparities among Black Americans compared to non-Hispanic (NH) White Americans.^2,16-19^ These studies have either used area-level racial and ethnic makeup alongside aggregated credit panel data^19-22^ or used survey data, where respondents self-report their race, ethnicity, and medical debt.^2,16-18^ Because aggregate analyses use area-level data, they may be prone to ecological biases and obscure within-area differences in medical debt. Analyses of survey data reports of medical debt may be prone to underreporting due to social desirability bias and because individuals may not accurately estimate the amount of medical debt they have. Analyses of borrower-level credit panel data would improve the reliability of any assessment of racial and ethnic disparities in medical debt.

Evidence about how policies and interventions may impact disparities is nascent. One broader health policy that can address debt is Medicaid expansion.^20,23-25^ Low-income patients experience resource constraints that restrict their ability to pay for medical bills and push them to choose to go uninsured or coverage under high cost-sharing plans. Prior evidence suggests that Medicaid expansion can reduce debt and racial and ethnic differences in insurance coverage.^26^ Past research indicates that US states that expanded Medicaid experienced reductions in medical debt compared to states that did not expand Medicaid,^20,23-25^ but these studies did not examine its effect by race and ethnicity.

We use a nationally representative sample of credit report data from 2016 to 2022 to estimate racial and ethnic differences in medical debt in collections among non-elderly adults. Then, we examine medical debt and disparities in medical debt based on Medicaid expansion status.

## Methods

### Data sources and study sample

We analyze data from the University of California Consumer Credit Panel (UC-CCP) from March 2016 to March 2022, or just before changes made to medical debt reporting practices. The UC-CCP's national sample provides nationally representative information for 2% of borrowers in the United States from 1 major credit bureau.

This data includes all debts reported to the credit bureau, including debt amount and source (eg, medical vs credit card debt). We include borrowers for whom imputed race and ethnicity information was available in the UC-CCP's Q1 archive from 2016 to 2022. Our analytic sample includes 46 states and Washington DC, since 4 states (CO, CT, VA, and UT) have privacy laws that limit the imputation of race and ethnicity. The bureau provided imputed racial and ethnic information for ∼25% of borrowers in each Q1 archive from 2016 to 2022. Appendix SA1 includes information about the sample selection.

We include only borrowers ages 18-64 to study debt among working age adults. The analytic sample includes 23 093 888 borrowers from 1 of the 4 BISG-reliable racial and ethnic groups, representing 16.1% of borrowers. Of these borrowers, 64.6% (*n* = 14 922 725) were NH White, 11.4% (*n* = 2 641 009) were NH Black, 6% (*n* = 1 376 479) were NH Asian or Pacific Islander, and 18% (*n* = 4 153 675) were Hispanic.

### Measures

#### Imputed race and ethnicity

Credit report data do not include borrower race and ethnicity. For this research, the credit bureau used a validated imputation method, RAND's Bayesian Imputed Surname Geocoding (BISG) method, to impute the race and ethnicity of each borrower based on geocoded address and surname. BISG is a widely used method for imputing race and ethnicity.^14,27-30^ BISG-produced estimates are considered most reliable for NH White; NH Black; NH Asian and Pacific Islander (API); and Hispanic populations.^27^ Predictive accuracy for these groups is high across these groups, with concordance statistics of 0.95 for Hispanic borrowers, 0.94 for API borrowers, and 0.93 for NH White and Black borrowers.^27^

We use the “single classification” approach, where borrowers are classified based on the highest racial and ethnic probability. To break ties, we randomly assigned borrowers to one of these tying categories (*n* = 2752 or 0.01% of the analytic sample).

#### Outcomes

We focus on medical debt that is reported to be in collections or purchased by a debt buyer. For more information about our identification of medical debt in the credit panel, see Appendix SA2. We adjust all amounts of debt for inflation using the consumer price index for medical care from the Federal Reserve Bank of St. Louis. In this study, our primary outcome is the annual “flow” of medical debt. Our secondary outcome is the “stock” of medical debt.

Annual flow of medical debt was the amount of medical debt in collections opened in the past year for each borrower.^20,31,32^ We examine unconditional mean of medical debt, the percentage of borrowers with any non-zero debt, then the mean amount of debt conditional on having any debt.

The “stock” of medical debt is defined as the total balance of outstanding medical debt in collections owed by each borrower. This measure includes the balance of all debts included in the annual flow measure, as well as the balance of all other outstanding medical debt in collections reported to the credit bureau. Stock is considered a secondary measure in our study because amounts can change for reasons beyond payment of debt, as collectors face restrictions in initial reporting of debt but are not required to continue reporting debt for any given period of time.^31,32^ We include stock to align with prior research's inclusion of the measure to provide a comprehensive view of medical debt.^20^

### Analyses

For each racial and ethnic group for which RAND BISG is considered reliable,^27^ we examine our measures for both the annual flow and stock outcomes, obtain 95% confidence intervals, and plot trends over time.

We conduct 3 sensitivity analyses. First, we compare estimates of medical debt when using BISG predicted probabilities as weights compared to our main specification using the single classification approach. Second, to address concerns that missingness may correlate with study outcomes, we compare borrowers with missing vs non-missing imputed racial and ethnic information across study outcomes. Third, to assess the potential confounding impact of income on our main results, we assign borrowers to a decile of per capita income from the 2017 to 2021 American Community Survey and plot our outcomes. However, because past research has shown that members of racial and ethnic minoritized groups living in wealthy and highly educated areas are most likely to be misclassified as NH White by BISG,^33^ we consider this analysis exploratory. All these analyses were conducted using R.

To compare trajectories in debt by racial and ethnic groups by Medicaid expansions status, we consider 3 groups based on dates of Medicaid expansion: expansion before March 2016 (*n* = 28 + DC), during our study period (*n* = 6), and without expansion before March 2022 (*n* = 12). For more information on which states fell into each group, see Appendix SA3.

To enable comparisons between states that expanded Medicaid vs those that did not, we use a regression model predicting (1) probability of having any medical debt (using logit models) and (2) amount of medical debt (using linear models). Our model includes an interaction term between year, racial and ethnic group, and Medicaid expansion status as well as their individual terms for these covariates. We then plot predictions by year for each racial and ethnic group, stratified by expansion status. We used Stata 17 for these analyses.

This project was deemed as not human subjects research by the University of California, Berkeley Committee for Protection of Human Subjects.

## Results

### Overall descriptives

#### Annual flow

The mean annual flow of medical debt in collections declined from $115 in 2016 [95% CI, 114-116] to $97 [95% CI, $96-98] in 2022 (Appendix SA4). Mean annual flow of medical debt was relatively stable from 2016 to 2018, then steadily climbed to a peak amount in the Q1 2020 archive. In 2021 and 2022, medical debt in collections fell. Throughout this period, NH Black borrowers had the highest mean annual flow of medical debt ($201 [95% CI, $197-205] in 2016, then $171 [95% CI, $167-174] in 2022). Hispanic borrowers had the second highest mean annual flows ($114 [95% CI, $112-117] in 2016, then $106 [95% CI, $104-109] in 2022), followed by NH White borrowers ($108 [95% CI, $107-109] in 2016, then $90 [95% CI, $88-91] in 2022). Non-Hispanic API borrowers had persistently the lowest mean annual flow of medical debt in collections of all groups ($19 [95% CI, $17-21] in 2016, then $15 [95% CI, $13-16] in 2022).

##### Percent of borrowers with any annual flow

**Figure 1:**
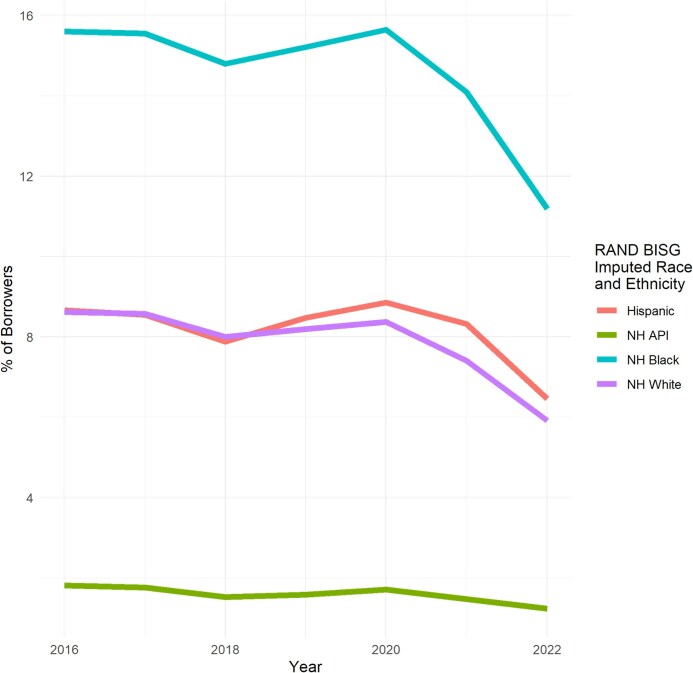
Percentage of borrowers with any annual flow of medical debt by imputed race and ethnicity. Source: Authors’ analysis of data from the University of California Consumer Credit Panel (UC-CCP), 2016-2022. NH, non-Hispanic, BISG, Bayesian Imputed Surname Geocoding.

Overall, the percentage of borrowers with any amount of annual flow of medical debt in collections was 9% [95% CI, 9%-9.1%] in 2016 and declined to 6.3% [95% CI, 6.3%-6.3%] in 2022. We show the percentage of borrowers with any annual flow by imputed race and ethnicity in Figure 1.

Non-Hispanic Black borrowers had the highest proportion of borrowers with any annual flow of medical debt in collections (15.6% [95% CI, 15.5%-15.7%] in 2016, then 11.2% [95% CI, 11.1%-11.3%] in 2022). Hispanic borrowers had the second highest percentage of borrowers with any annual flow of medical debt (8.7% [95% CI, 8.6%-8.7%] in 2016, then 6.5% [95% CI, 6.4%-6.5%] in 2022), followed by non-Hispanic White borrowers (8.6% [95% CI, 8.6%-8.7%] in 2016, then 5.9% [95% CI, 5.9%-6%] in 2022). Non-Hispanic API borrowers had the lowest percentage of borrowers with any non-zero annual flow (1.8% [95% CI, 1.8%-1.9%] in 2016, then 1.2% [95% CI, 1.2%-1.3%] in 2022).

##### Mean annual flow conditional on having any annual flow of debt

**Figure 2:**
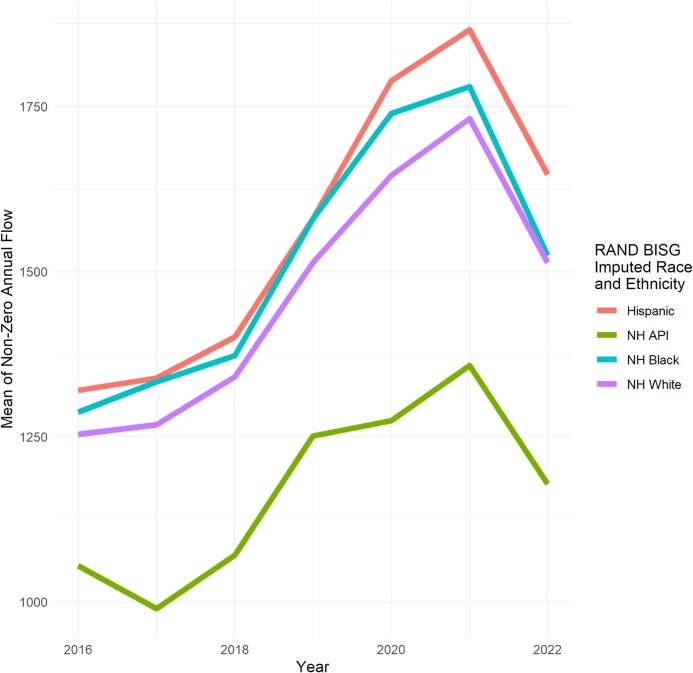
Caption: Mean of annual flow of medical debt (conditional on having any annual flow) by imputed race and ethnicity. Source: Authors’ analysis of data from the University of California Consumer Credit Panel (UC-CCP), 2016-2022. NH, non-Hispanic; BISG, Bayesian Imputed Surname Geocoding.

Among borrowers with any non-zero annual flow, the mean amount of annual flow was $1269 [95% CI, $1257−$1280] in 2016. Mean debt among borrowers with any non-zero annual flow increased from 2016 to 2021 before falling slightly in 2022 ($1538 [95% CI, $1523-1553]), which still represented an increase compared to 2016 levels. We show the mean annual flow among borrowers with any annual flow by imputed race and ethnicity in Figure 2.

There were small gaps between Hispanic, non-Hispanic Black, and NH White borrowers for annual flow conditional of having non-zero flow of medical debt. Hispanic borrowers had the highest mean of non-zero annual flow ($1320 [95% CI, $1291-1349] in 2016, then $1647 [95% CI, $1612-$1681] in 2022). Non-Hispanic Black borrowers had the second highest mean of non-zero annual flow ($1287 [95% CI, $1263-1312] in 2016, then $1524 [95% CI, $1493-1556] in 2022). Non-Hispanic White borrowers had the third highest mean of non-zero annual flow ($1254 [95% CI, $1239-$1268] in 2016, then $1513 [95% CI, $1494-1532] in 2022).

Non-Hispanic API borrowers had the lowest mean annual flow among borrowers with any non-zero flow of medical debt ($1055 [95% CI, $944-1165] in 2016, then $1178 [95% CI, $1494-1532] in 2022).

#### Stock

Across all borrowers, mean stock was $442 [95% CI, $439-445] in 2016 and fell to $308 [95% CI, $305-310] in 2022 (Appendix SA5). Non-Hispanic Black borrowers had the highest mean stock ($679 [95% CI, $669-689] in 2016, then $498 [95% CI, $490-507] in 2022). Hispanic borrowers had the second highest mean stock ($457 [95% CI, $449-464] in 2016, then $317 [95% CI, $312-323] in 2022), though this was close to the mean stock among NH White borrowers ($426 [95% CI, $422-430], then $297 [95% CI, $294-299] in 2022). Non-Hispanic API borrowers had the lowest mean stock ($84 [95% CI, $78-90], then $53 [95% CI, $50-57] in 2022).

##### Percent of borrowers with any stock

**Figure 3:**
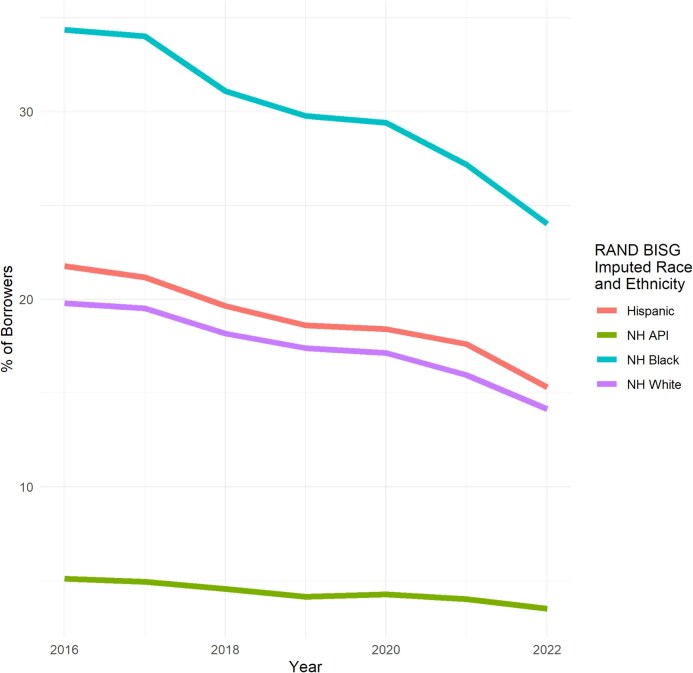
Caption: Percentage of borrowers with any stock of medical debt by imputed race and ethnicity. Source: Authors’ analysis of data from the University of California Consumer Credit Panel (UC-CCP), 2016-2022. NH, non-Hispanic; BISG, Bayesian Imputed Surname Geocoding.

Overall, the percentage of borrowers with any stock of medical debt in collections was 21% [95% CI, 21%-21%] in 2016 and declined to 14.8% [95% CI, 14.8%-14.8%] in 2022. We show the percent of borrowers with any stock by imputed race and ethnicity in Figure 3.

Non-Hispanic Black borrowers had the highest percentage of borrowers with any stock of medical debt in collections (34.4% [95% CI, 34.2%-34.5%] in 2016, then 24% [95% CI, 23.9%-24.1%] in 2022). Hispanic borrowers had the second highest percentage of borrowers with any stock of medical debt in collections (21.8% [95% CI, 21.7%-21.9%] in 2016, then 15.3% [95% CI, 15.2%-15.4%] in 2022), followed closely by NH White borrowers (19.8% [95% CI, 19.7%-19.8%] in 2016, then 14.1% [95% CI, 14.1%-14.2%] in 2022). Non-Hispanic API borrowers had the lowest percentage of borrowers with any stock of medical debt in collections (5.1% [95% CI, 5%-5.2%] in 2016, then 3.5% [95% CI, 3.4%-3.6%] in 2022).

##### Mean stock conditional on having any stock of debt

**Figure 4:**
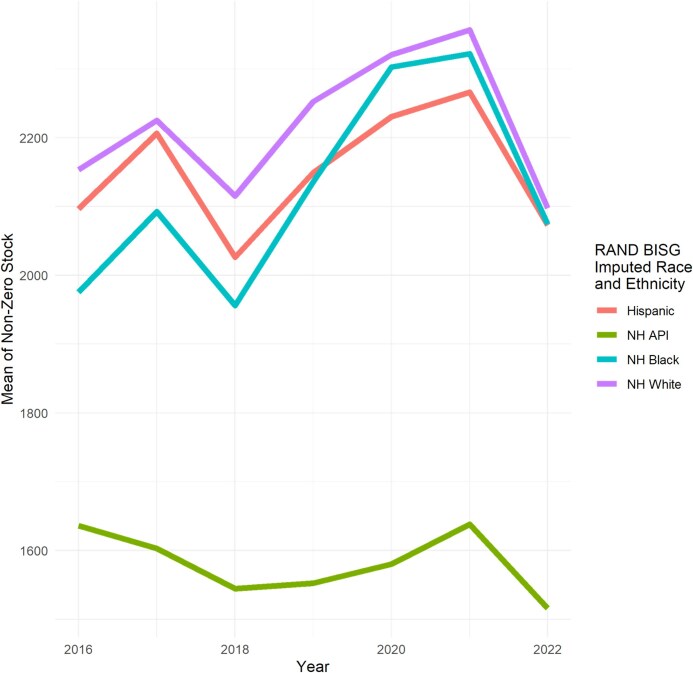
Caption: Mean of stock of medical debt (conditional on having any stock) by imputed race and ethnicity. Source: Authors’ analysis of data from the University of California Consumer Credit Panel (UC-CCP), 2016-2022. NH, non-Hispanic; BISG, Bayesian Imputed Surname Geocoding.

Among borrowers with any non-zero stock, the mean amount was $2103 [95% CI, $2089-2117] in 2016. Similar to mean annual flow conditional on having any non-zero medical debt, mean debt among borrowers with any non-zero stock increased from 2016 to 2021 before falling slightly in 2022 ($2080 [95% CI, $2065-2094]), which still represented an increase compared to 2016 levels. We show the mean stock among borrowers with any stock by imputed race and ethnicity in Figure 4.

In 2016, White borrowers had the highest mean non-zero stock ($2153 [95% CI, $2135-2171]), Hispanic borrowers had the second highest mean non-zero stock ($2097 [95% CI, $2063-2130]), while Black borrowers had the third highest ($1976 [95% CI, $1947-2004]). By 2022, there were relatively small gaps between NH White, NH Black, and Hispanic borrowers for mean stock among borrowers with non-zero stock of medical debt (NH White: $2097 [95% CI, $2078-2116]; NH Black: $2074 [95% CI, $2041-2108]; Hispanic: $2073 [95% CI, $2040-$2106]).

Non-Hispanic API borrowers had the lowest non-zero stock of medical debt across time periods ($1636 [95% CI, $1524-1748] in 2016, then $1516 [95% CI, $1413-1619] in 2022).

#### Medicaid expansion

We plot the outcomes by Medicaid expansion status in Appendix SA6, with predicted probabilities and amounts are available in Appendix SA7. In non-expansion states, NH Black adults faced the highest probability of medical debt, peaking at 0.21 in 2020 before falling to 0.15 by 2022. Probabilities for Hispanic and NH White adults remained below 0.15, while NH API individuals stayed under 0.04. Comparatively, expansion states showed lower probabilities across all demographics. 2016 mean flows in non-expansion states were similar for NH Black, Hispanic, and NH White borrowers, while NH API borrowers were lowest. These amounts rose through 2021 before a 2022 decline, maintaining a consistent gap for NH API groups. Expansion states mirrored these trends but with significantly lower mean flows and narrower differences by racial and ethnic group, ranging from $932 (NH API) to $1059. Similar to non-expansion states, borrowers in expansion states experienced increases in amount of flow up to 2021 before declining in 2022.

Similar trends existed for predicted probabilities in stock of debt (Appendices SA6 and SA7). However, states that expanded Medicaid had declining amounts of stock across racial and ethnic groups from 2016 to 2022, whereas non-expansion states experienced increases until 2021 before declining in 2022.

States that expanded Medicaid over our study period had lower mean flow and stock by the end of the study period across all racial and ethnic groups (Appendix SA6). This decline was driven by a decrease in the percentage of borrowers reporting any annual flow or stock across all racial and ethnic groups. The mean annual flow among borrowers with any flow increased among NH Black and White borrowers, while it decreased for NH API and Hispanic borrowers. Non-Hispanic White borrowers had similar increases in mean stock among borrowers with any stock, while the other racial and ethnic groups experienced declines.

### Sensitivity analysis

Analyses using RAND BISG predicted probabilities as weights are presented in Appendix SA8. While levels of debt varied slightly across the 2 approaches, differences between racial and ethnic groups were consistent across approaches across outcomes. This suggests risk of misclassification of borrowers in our main analyses is unlikely to change our main findings and conclusions.

Analyses examining differences in study outcomes by missingness of imputed race and ethnicity are shown in Appendix SA9. Borrowers with missing data had slightly lower levels of medical debt but followed the same trends over time as borrowers with imputed racial and ethnic data. Subsequently, it is likely the main results reflect higher levels of debt than if all borrowers were analyzed.

The results for each analysis stratified by area-level income decile are in Appendix SA10. For both the mean annual flow and stock, amounts are highest among borrowers living in middle-income deciles 4 through 7 across racial and ethnic groups. Similar to findings from the main analyses, the differences in per capita medical debt are driven by differences in the percentage of borrowers with any medical debt and the mean amount owed by those borrowers.

Within each income decile and across both flow and stock, NH Black borrowers consistently experience the highest levels of medical debt while NH API borrowers experience the lowest levels. NH White and Hispanic borrowers consistently have higher debt levels than NH API borrowers but lower levels than NH Black borrowers. Depending on the measure and income decile, Hispanic borrowers have higher or lower debt levels than NH White borrowers; for example, for mean annual flow, Hispanic borrowers have higher flow to income decile 5, then NH White borrowers have higher levels from income decile 6 onwards.

## Discussion

Medical debt is a pervasive financial burden for Americans and is associated with worse health and financial outcomes. We found that NH Black borrowers faced the highest levels of medical debt. Hispanic borrowers tended to have comparable medical debt levels than NH White borrowers. Differences across groups were larger in the percentage of borrowers facing any debt vs the amount owed by each borrower.

Across all analyses, NH Black borrowers experienced increased medical debt compared to other racial and ethnic groups. These results represent a somber yet expected result of well-documented differences across health^34-36^ and finances.^37^ Importantly, medical debt may also be a part of a cyclical relationship whereby medical debt contributes to both worse health and overall finances. When medical debt is accrued, it may lead to further barriers to health-promoting activities and increase financial instability. In these ways, our results reflect a stark consequence of and contributor to inequities across social domains that Black Americans face.^38,39^

We found comparable medical debt for Hispanic and NH White borrowers. This finding is consistent with some past research,^40^ but not others which show that Hispanic borrowers have a higher level of debt.^4^ At least some of the differences between this research and studies with divergent results may be due to definitions of medical debt (debt in collections vs debts paid over time to providers, on credit cards, owed to families, etc.). Further work to resolve these questions is thus warranted.

Similar to past research,^20,23-25^ we identified large differences in medical debt amongst racial and ethnic groups based on state-level Medicaid expansion status. States that implemented Medicaid expansion experienced declines in per capita flow and stock of medical debt, driven by declines in the overall percentage of borrowers reporting any debt across racial and ethnic groups. While states with expanded Medicaid experienced overall lower levels of medical debt across racial and ethnic groups, gaps between groups remained. Ultimately, while Medicaid expansion is associated with reduced levels of medical debt across racial and ethnic groups, it may not specifically eliminate medical debt disparities compared to NH White borrowers.

In this study, both overall levels of medical debt and differences between racial and ethnic groups continued pre-COVID trends of decline, aligning with prior evidence.^19,41^ Several COVID era policies were implemented that may affect individuals’ risk of incurring medical debt, including continuous enrollment in Medicaid, increased subsidies for the health insurance exchanges, the shielding of patients from balance billing related to COVID-19 care and provision of funds from the government to cover care for uninsured patients via the CARES Act.^19,41^ In addition, many local governments used American Rescue Plan funds to directly cancel medical debt.^42^ Finally, it is possible stimulus payments may have increased household financial security, helping borrowers avoid medical debt. Despite concerns about potential increases in medical debt related to COVID-19 infection and subsequent need for care, these policies may have counteracted increases in medical debt, yielding the declines identified in this study.

While we identify critical differences in medical debt by race and ethnicity in this study, further research is needed to explore the mechanisms that produce the differences in medical debt by racial and ethnic group we identify in this study in order to fully understand the relevant policies that can narrow these disparities. Racial and ethnic disparities can be driven by any combination of income, utilization, healthcare prices, and collection practices, among others;^36,38,43^ understanding the most impactful policy solutions requires an assessment of which of these pathways may have more (or less) contribution to the accrual of medical debt. Linkage of credit panel data to other datasets that can be used to examine these mechanisms are thus important; linkage to tax returns could yield important insights regarding income and medical debt, and linkage to admissions and claims datasets could yield information regarding prices and utilization.

Beyond studying mechanisms to inform policy, another avenue of research includes expanding research specifically examining the impact of policies targeted at medical debt to the impact on disparities by race and ethnicity. Recent evidence includes studies on the effect of excluding medical debt from credit reports^44^ and additional consumer protections targeted at the provision of charity care and limiting collection activities.^45^ In addition, future research could examine the differential impact of COVID era policies on medical debt by race and ethnicity, especially given differences in frontline work and employment and local COVID policies.^46^ Understanding heterogeneity in effects of policies that target medical debt directly or impact pathways that drive debt is thus an important future direction for research and policy.

Study results should be considered given 4 limitations. First, because we used BISG, borrowers may be classified as a race and ethnicity that is different from their self-identified group. While we use weighted probabilities approach as a robustness check and our results were consistent, use of self-reported data is preferable. Second, RAND BISG estimates are available for a subset of borrowers. Without self-reported racial and ethnic data, it is not possible to assess whether borrowers with imputed racial and ethnic data are more or less representative than those without imputed race and ethnicity data. Third, medical debt in collections does not capture other forms of debt related to medical costs, like credit card debt, payday loans, debt owed to family or friends, payment plans set directly with the healthcare provider, or other extraordinary collection actions.^5^ Finally, we opted not to control for income in our main model because it mediates the relationship between race and medical debt. Our sensitivity analyses, which stratified the results by income deciles, however, found consistent results with the main models. This analysis is exploratory because of the misclassification risks of minoritized racial and ethnic borrowers who reside in high income areas.^33^

## Conclusion

The disproportionate medical debt level faced by Black borrowers compared to other racial and ethnic groups has endured over time. States that expanded Medicaid had lower overall levels of debt and smaller differences between racial and ethnic groups than states that did not. Results support interventions to directly address disparities in debt.

## Supplementary Material

qxag091_Supplementary_Data
