# Supplementary material for: Trends in medical debt in the United States by imputed borrower race and ethnicity, 2016-2022
Source: Health Aff Sch. 2026 May 22;4(5):qxag091. doi: 10.1093/haschl/qxag091 (PMC13196874; doi:10.1093/haschl/qxag091)
Supplement: qxag091_Supplementary_Data [file qxag091_supplementary_data.zip › appendix_v2.docx]

Trends in Medical Debt in the United States by Imputed Borrower Race and Ethnicity, 2016-2022

Online Supplement

Contents

[Appendix A1. Sample Selection Diagram 3](#_Toc226017605)

[Appendix A2. Defining Medical Debt in Collections 3](#_Toc226017606)

[Appendix A3. States by Medicaid Expansion 4](#_Toc226017607)

[Appendix A4. Mean of Annual Flow (All Borrowers) 5](#_Toc226017608)

[Appendix A5. Mean Stock (All Borrowers) 6](#_Toc226017609)

[Appendix A6. Descriptives for States Expanding Medicaid 7](#_Toc226017610)

[Mean annual flow 7](#_Toc226017611)

[Mean non-zero annual flow 9](#_Toc226017612)

[Percent of borrowers with any annual flow 10](#_Toc226017613)

[Mean stock 13](#_Toc226017614)

[Mean non-zero stock 15](#_Toc226017615)

[Percent of borrowers with any stock 17](#_Toc226017616)

[Appendix A7. Trends in Predicted Probabilities/Amounts for Each Racial and Ethnic Group by State Medicaid Expansion Status 19](#_Toc226017617)

[Percent of borrowers with any annual flow 19](#_Toc226017618)

[Mean non-zero annual flow 20](#_Toc226017619)

[Mean annual flow (all borrowers) 21](#_Toc226017620)

[Percent of borrowers with any stock 22](#_Toc226017621)

[Mean non-zero stock 23](#_Toc226017622)

[Mean stock (all borrowers) 23](#_Toc226017623)

[Appendix A8. Comparing Single Classification vs Weighted Approaches 25](#_Toc226017624)

[Mean annual flow (all borrowers) 25](#_Toc226017625)

[Mean non-zero annual flow 26](#_Toc226017626)

[Percent of borrowers with any annual flow 27](#_Toc226017627)

[Mean stock (all borrowers) 28](#_Toc226017628)

[Mean non-zero stock 29](#_Toc226017629)

[Percent of borrowers with any stock 30](#_Toc226017630)

[Appendix A9. Assessing Differences in Debt by Missingness of RAND BISG Imputed Race and Ethnicity, 2016-2022 30](#_Toc226017631)

[Appendix A10. Differences in Medical Debt Across Imputed Race and Ethnicity Stratified by Income Decile 35](#_Toc226017632)

[Annual flow 35](#_Toc226017633)

[Stock 37](#_Toc226017634)

## Appendix A1. Sample Selection Diagram


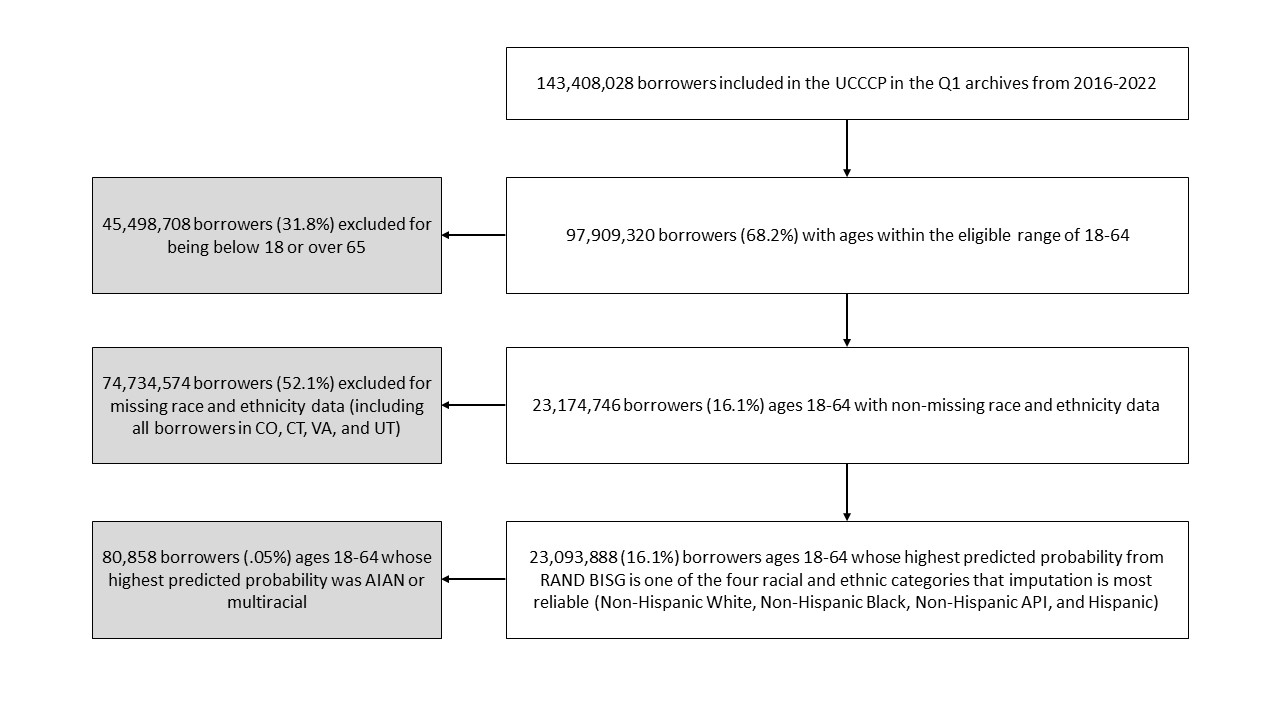


## Appendix A2. Defining Medical Debt in Collections

We take tradeline-level data and generate borrower-level measures of debt. To generate this measure, we have to identify when tradelines are 1) linked to medical debt, then 2) when such medical debt is in collections.

For 1), we consider tradelines as medical debt using fields generated by the credit panel providing access to this data. We identify tradelines as medical debt when:

- The originator class code variable is medical
- The enhanced account type variable is medical
- The key of business code is one of 13 subcodes belonging to the medical category

For 2), we use the loan type variable (as generated by CPL), where the value is either collection or debt buyer.

## Appendix A3. States by Medicaid Expansion

**Expanded Medicaid before March 2016 (28 + DC):** AK, AR, AZ, CA, DE, DC, HI, IL, IN, IA, KY, MD, MA, MI, MN, MT, NV, NH, NJ, NM, NY, ND, OH, OR, PA, RI, VT, WA, WV

**Did not expand Medicaid before March 2022 (12):** AL, FL, GA, KS, MS, NC, SC, SD, TN, TX, WI, WY

**Expanded Medicaid during study period (6):** LA (7/2016), ME(1/2019, retroactive to 7/2018), ID (1/2020), NE (10/2020), OK (7/2021), MO (10/2021, retroactive to 7/2021)

**Excluded because of RAND BISG (4):** CO, CT, VA, UT

## Appendix A4. Mean of Annual Flow (All Borrowers)


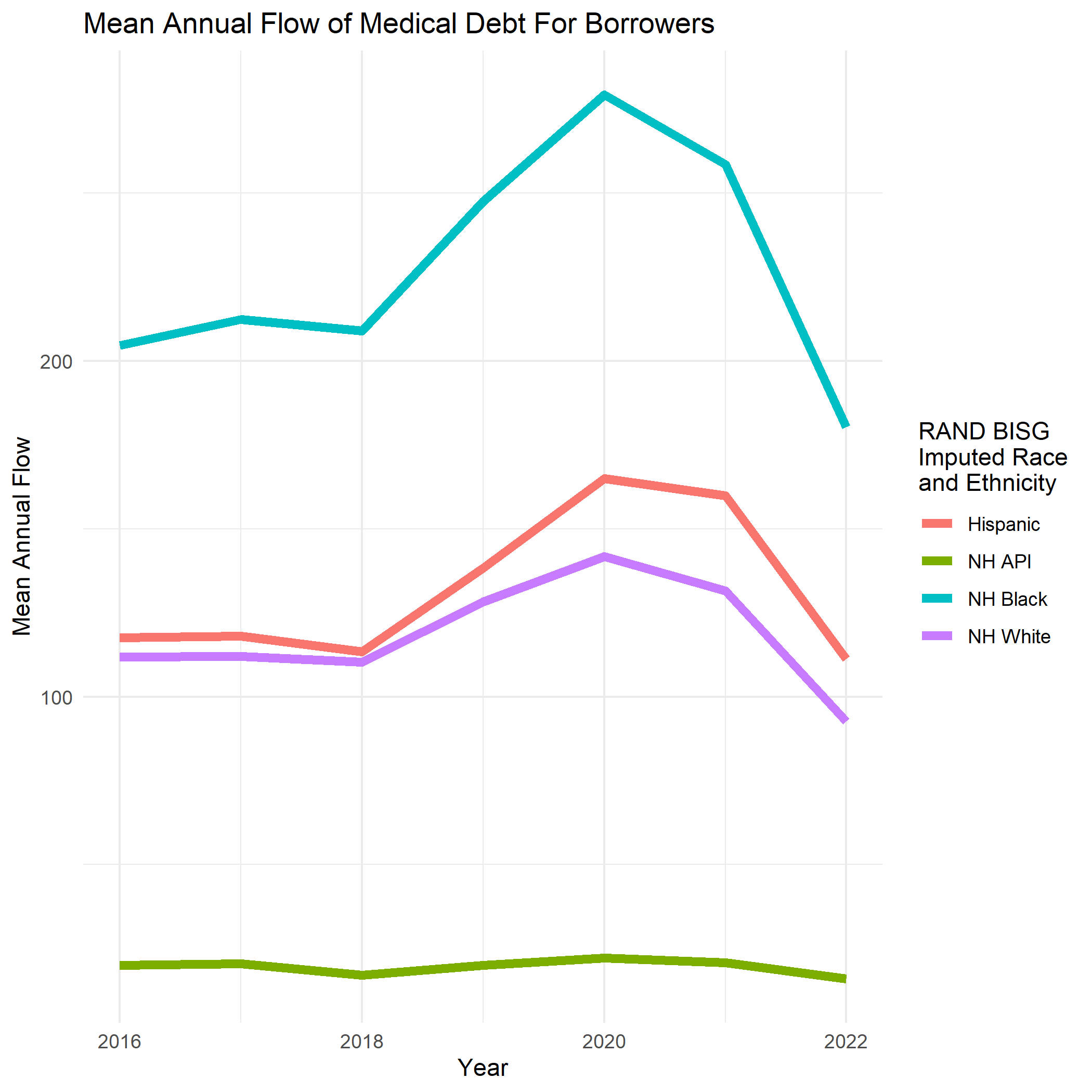


## Appendix A5. Mean Stock (All Borrowers)


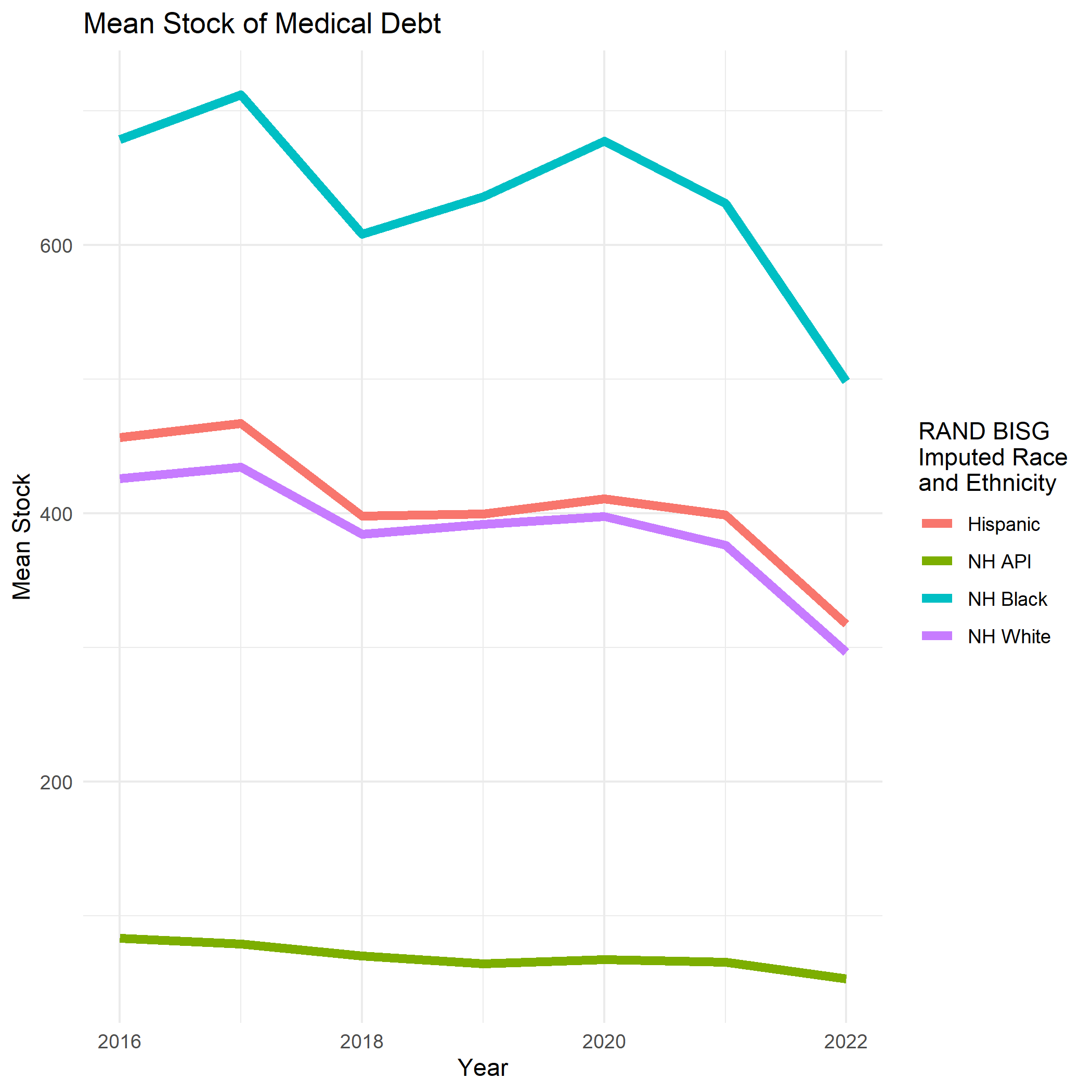


## Appendix A6. Descriptives for States Expanding Medicaid

### Mean annual flow

#### Consistent Medicaid Status


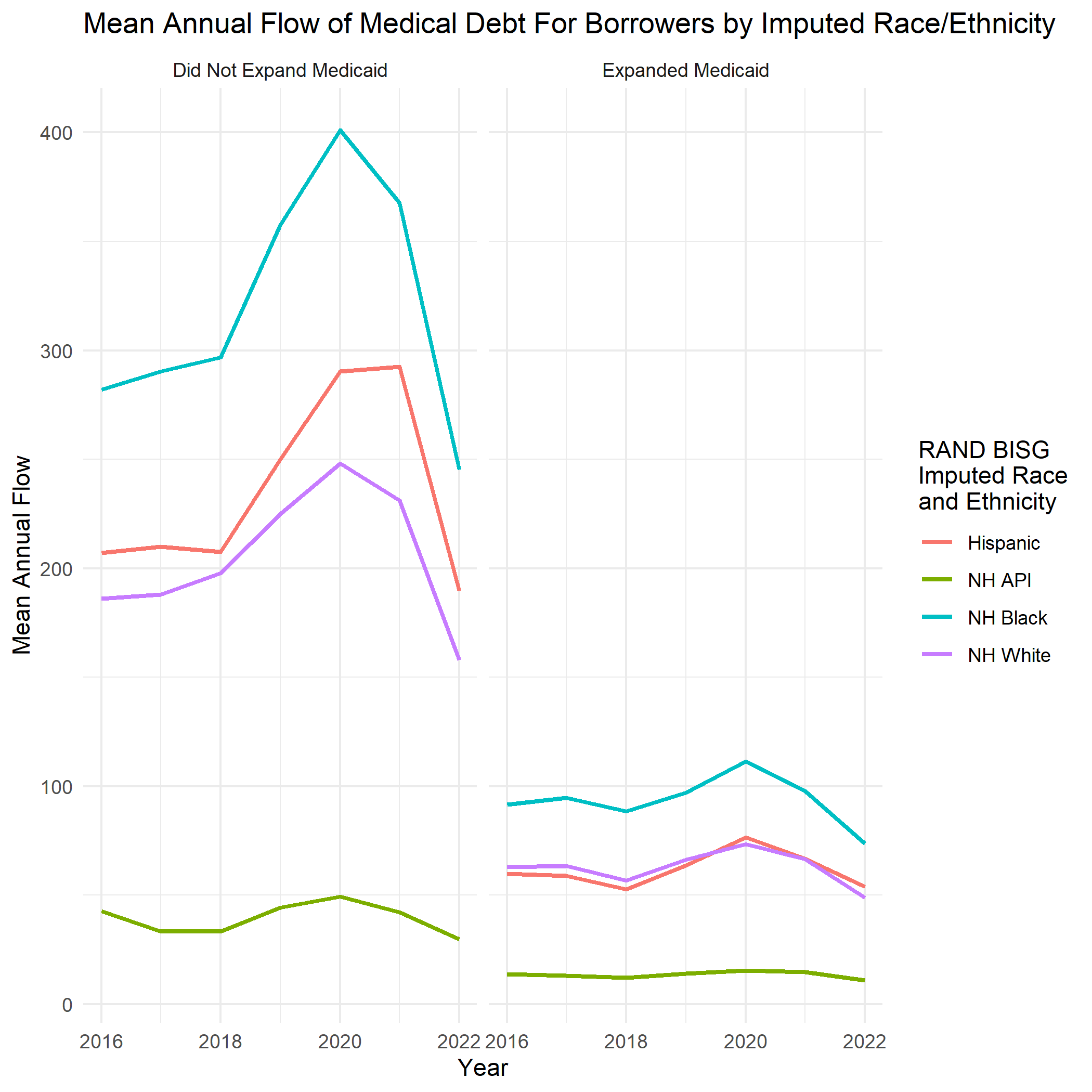


#### Implemented Medicaid from 2016-2022


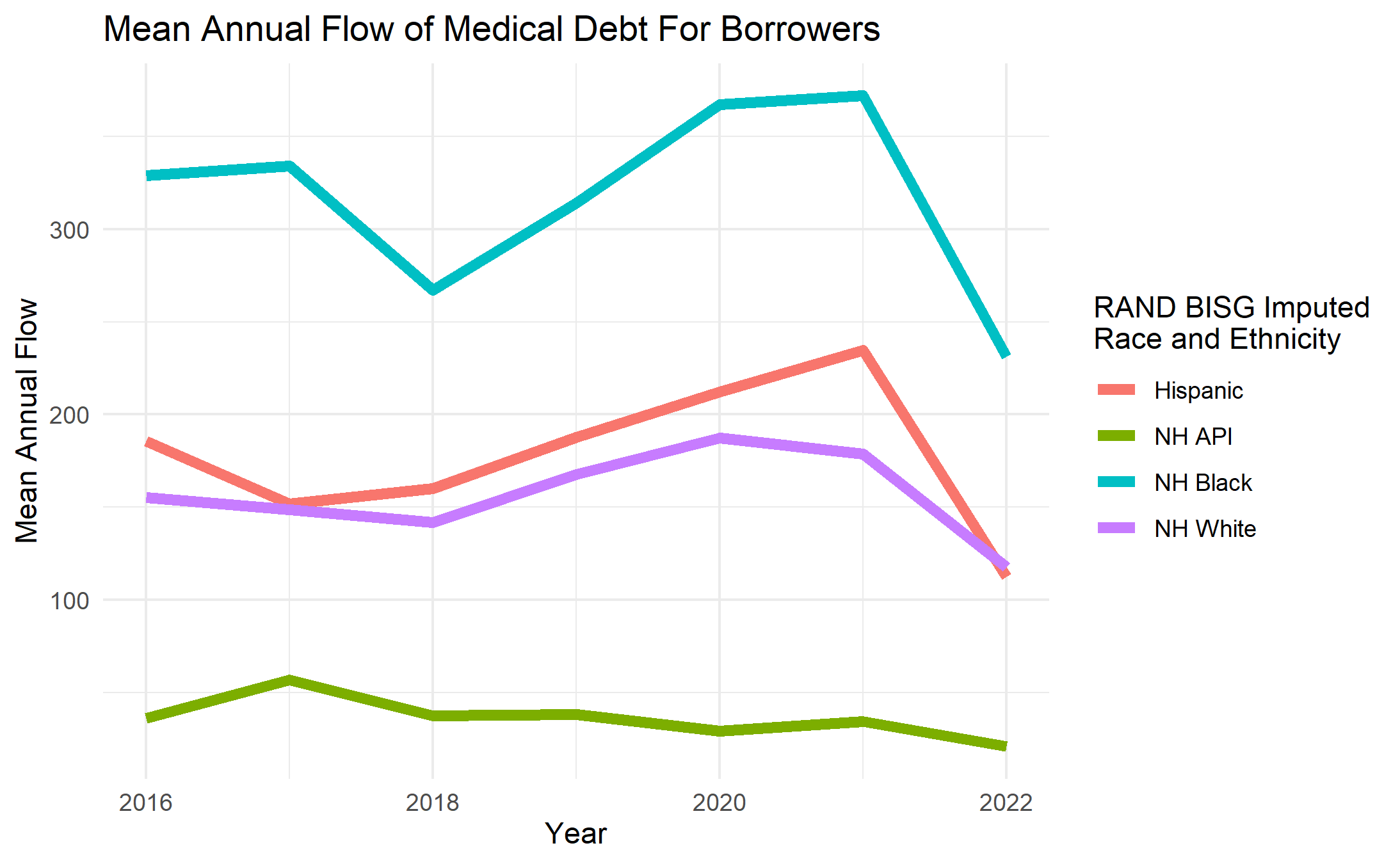


### Mean non-zero annual flow

#### Consistent Medicaid Status


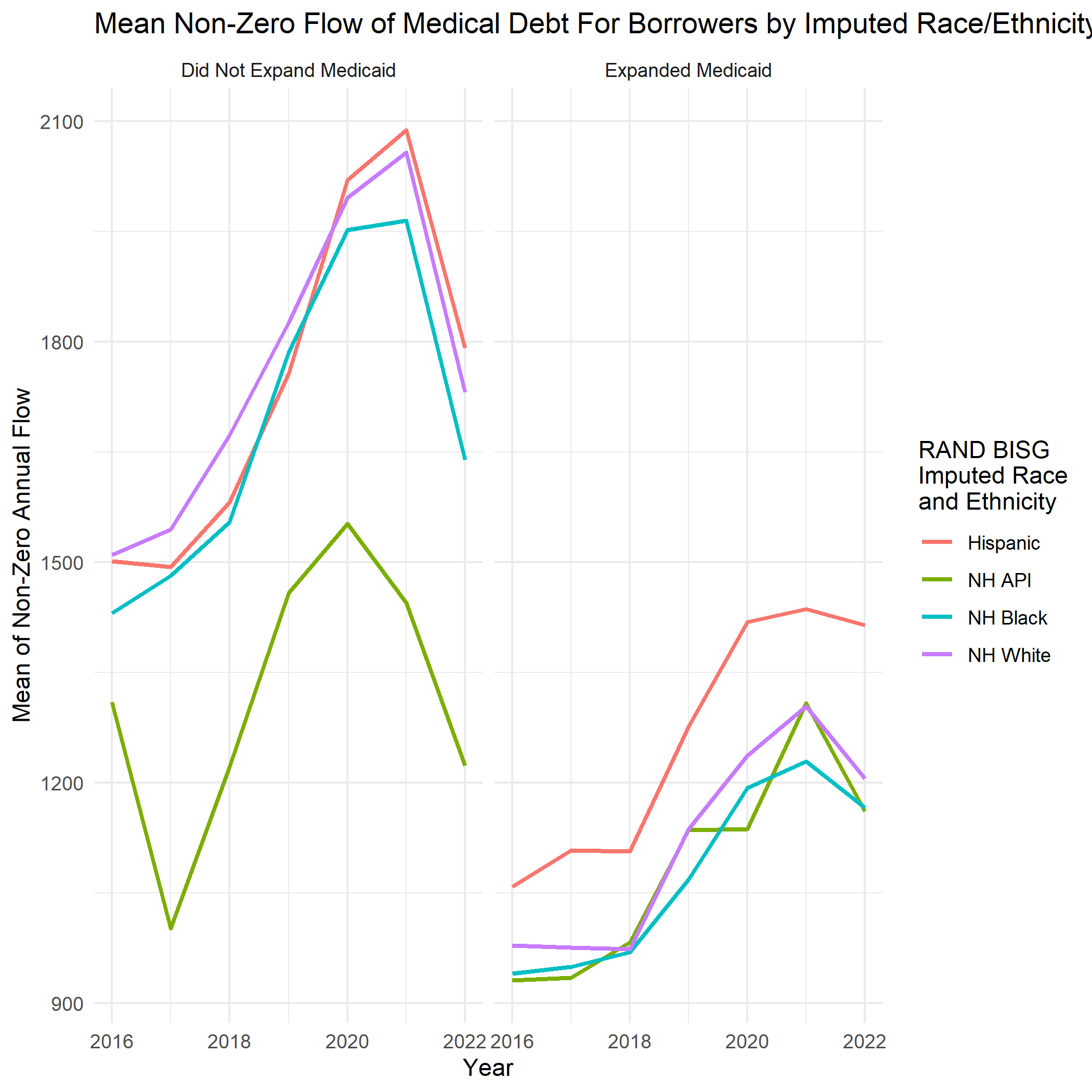


#### Implemented Medicaid from 2016-2022
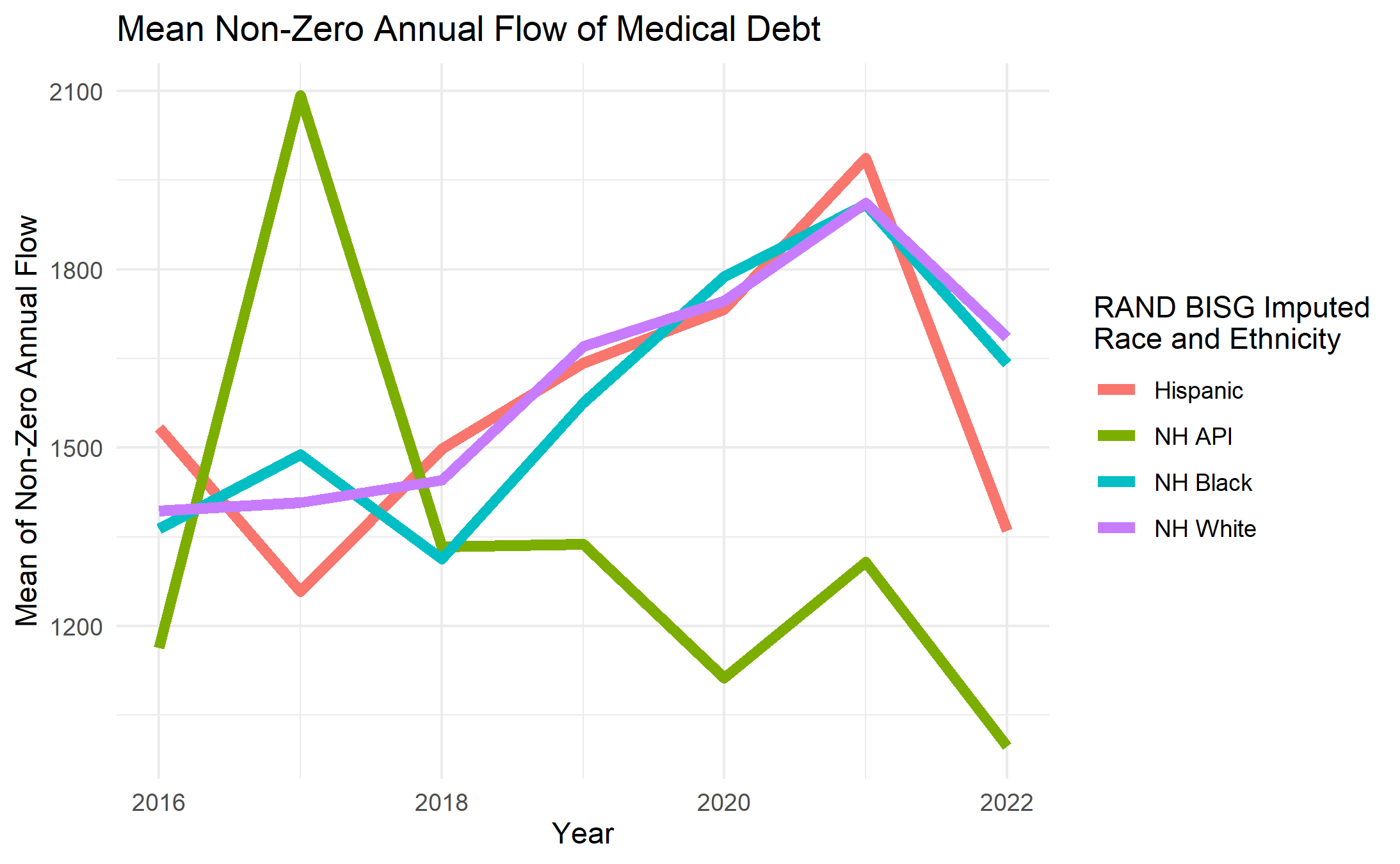


### Percent of borrowers with any annual flow

#### Consistent Medicaid Status


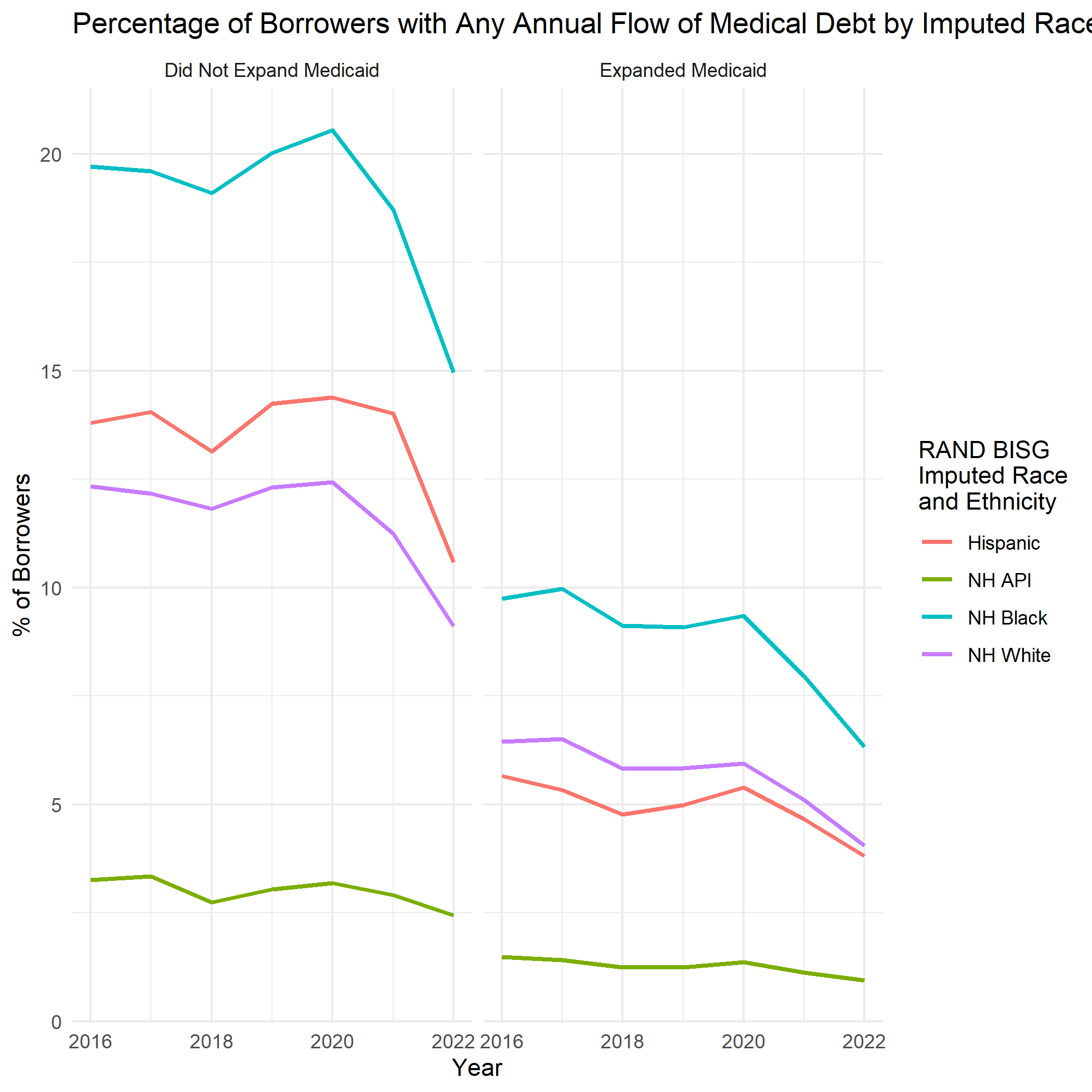


#### Implemented Medicaid from 2016-2022


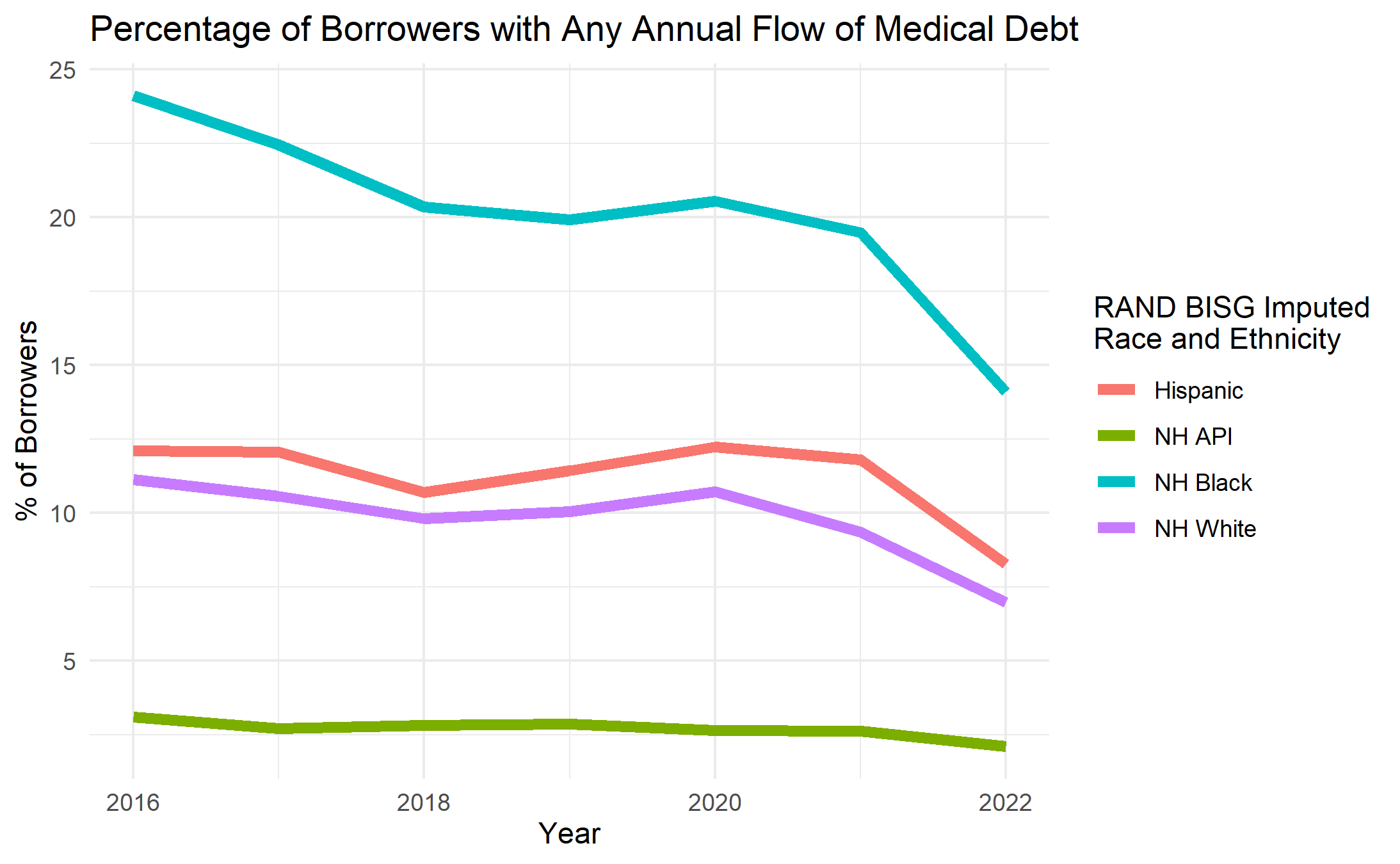


### Mean stock

#### Consistent Medicaid Status


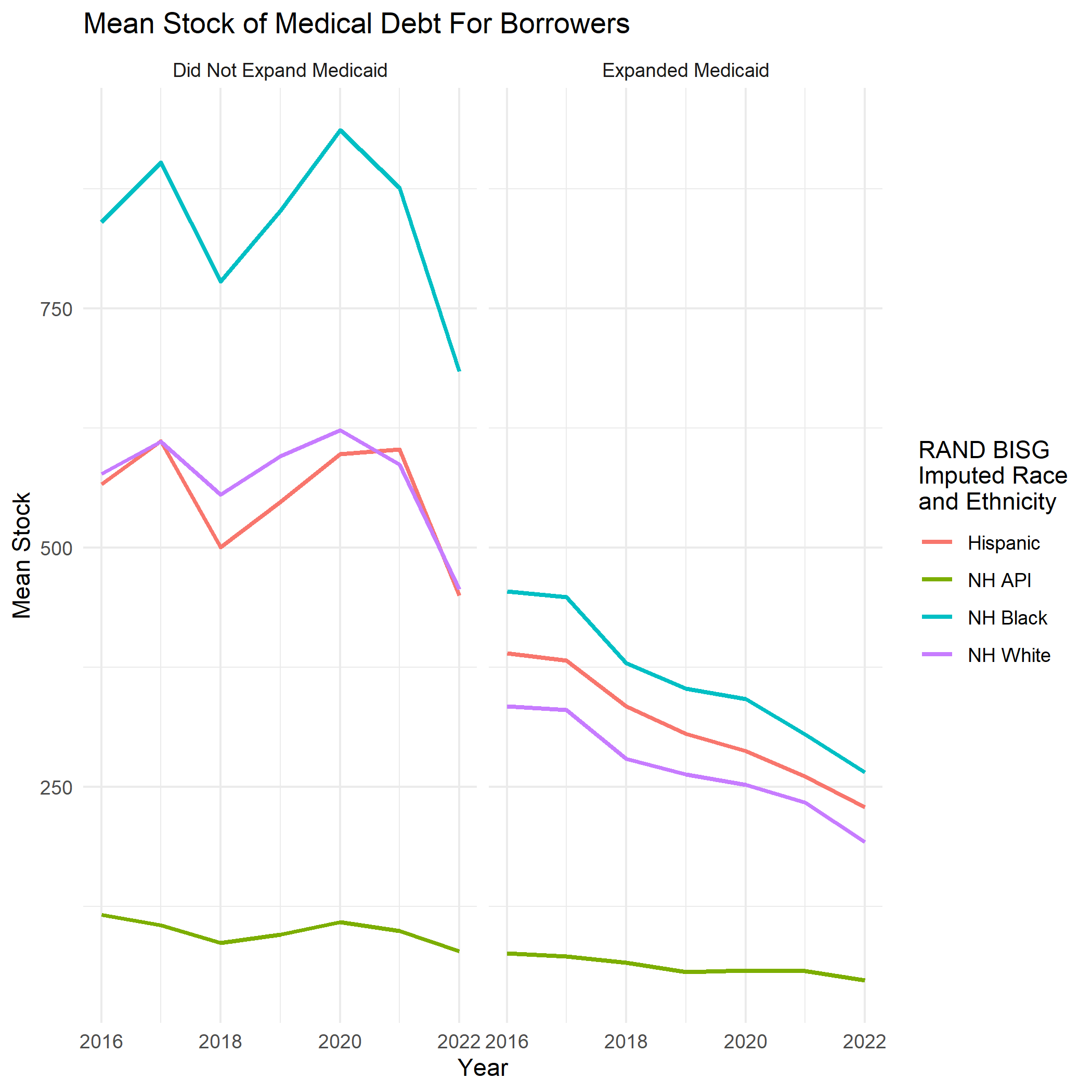


#### Implemented Medicaid from 2016-2022


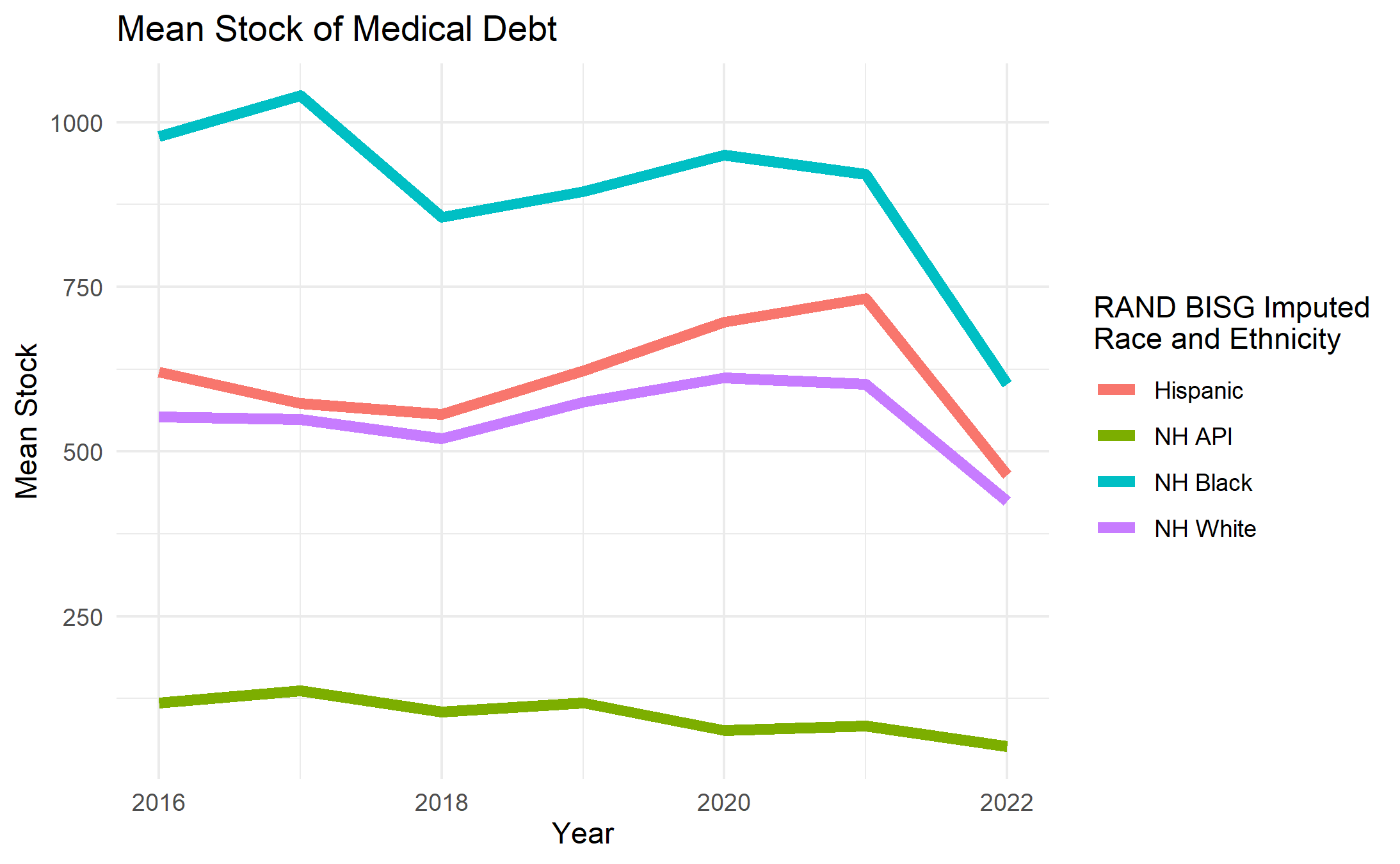


### Mean non-zero stock

#### Consistent Medicaid Status


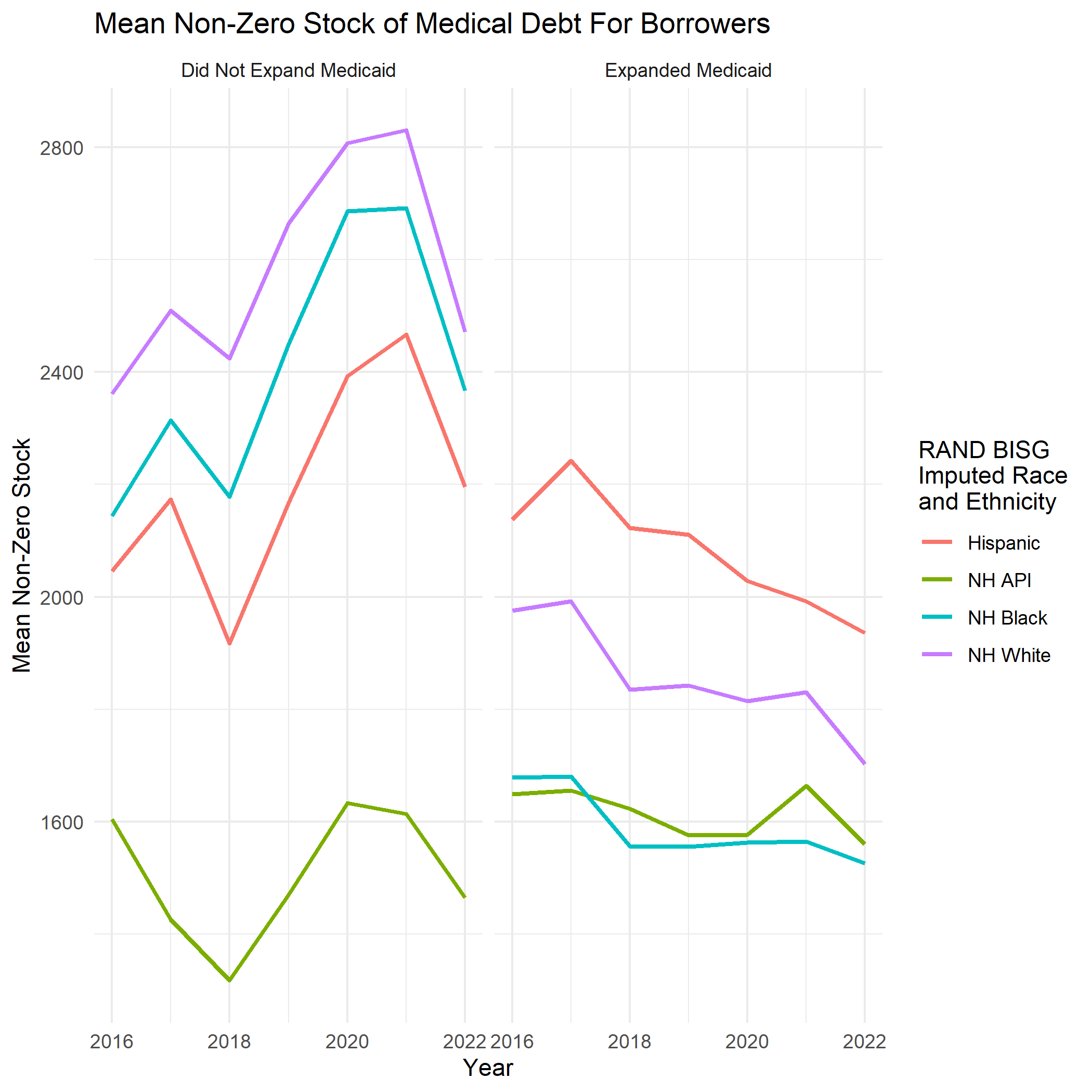


#### Implemented Medicaid from 2016-2022


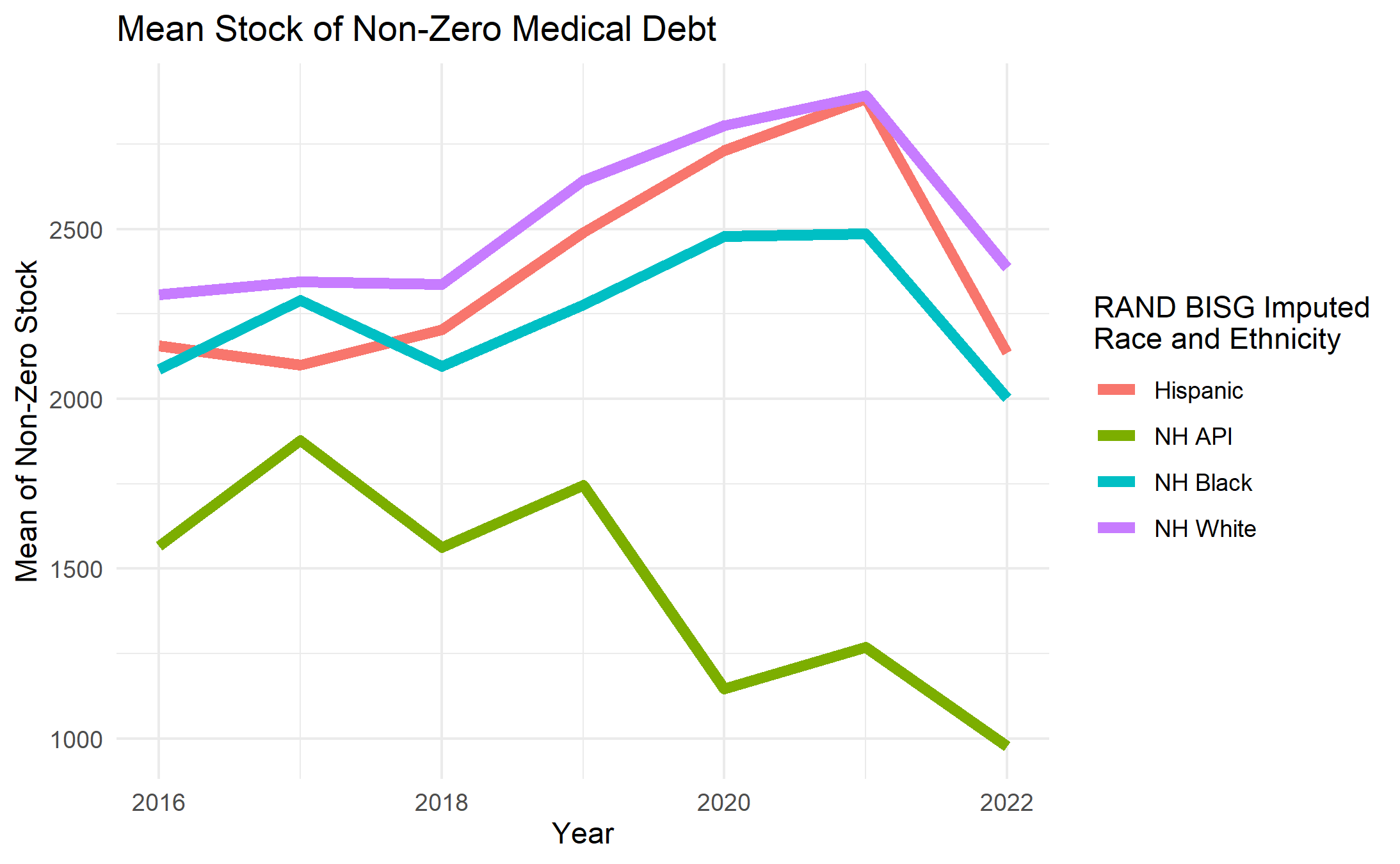


### Percent of borrowers with any stock

#### Consistent Medicaid Status


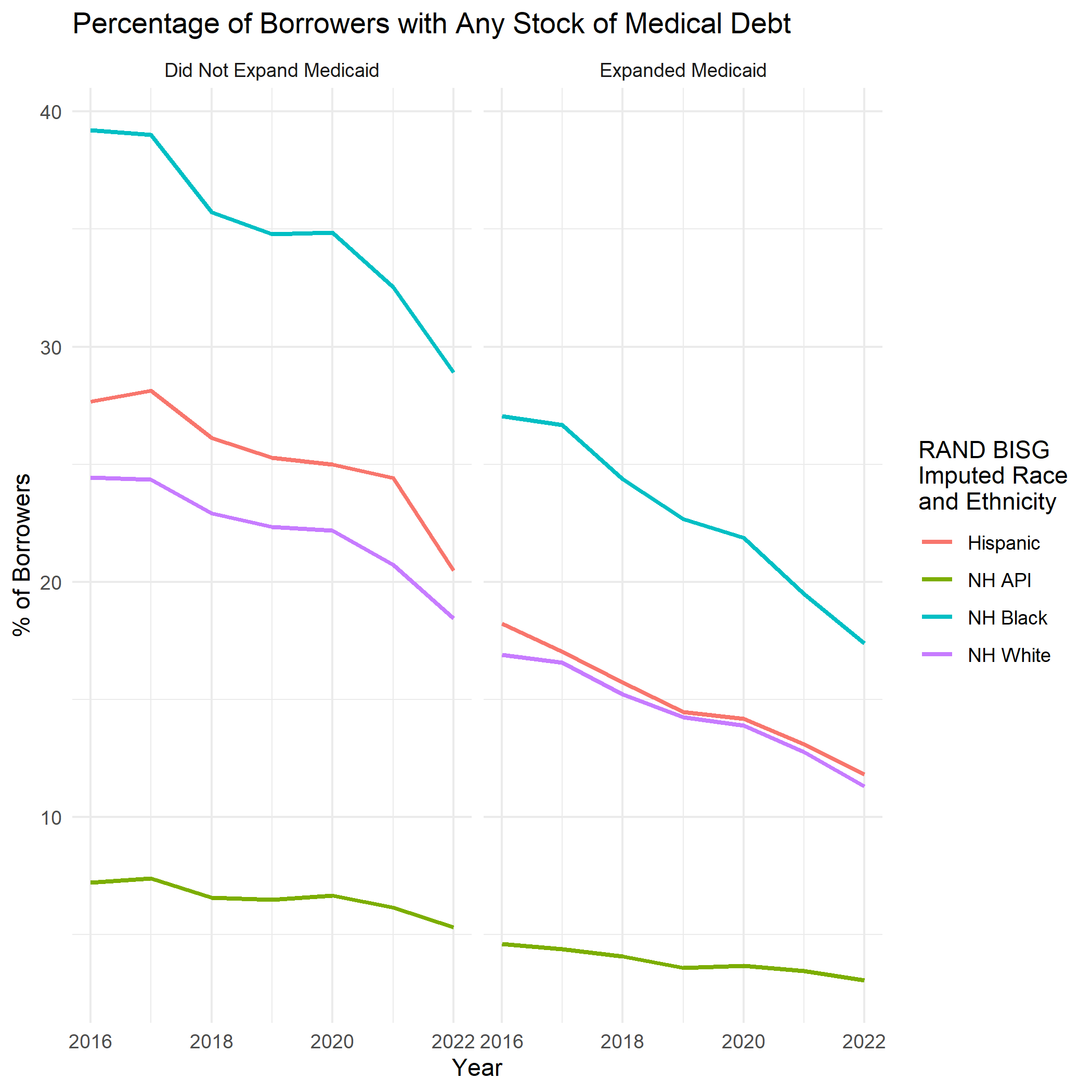


#### Implemented Medicaid from 2016-2022


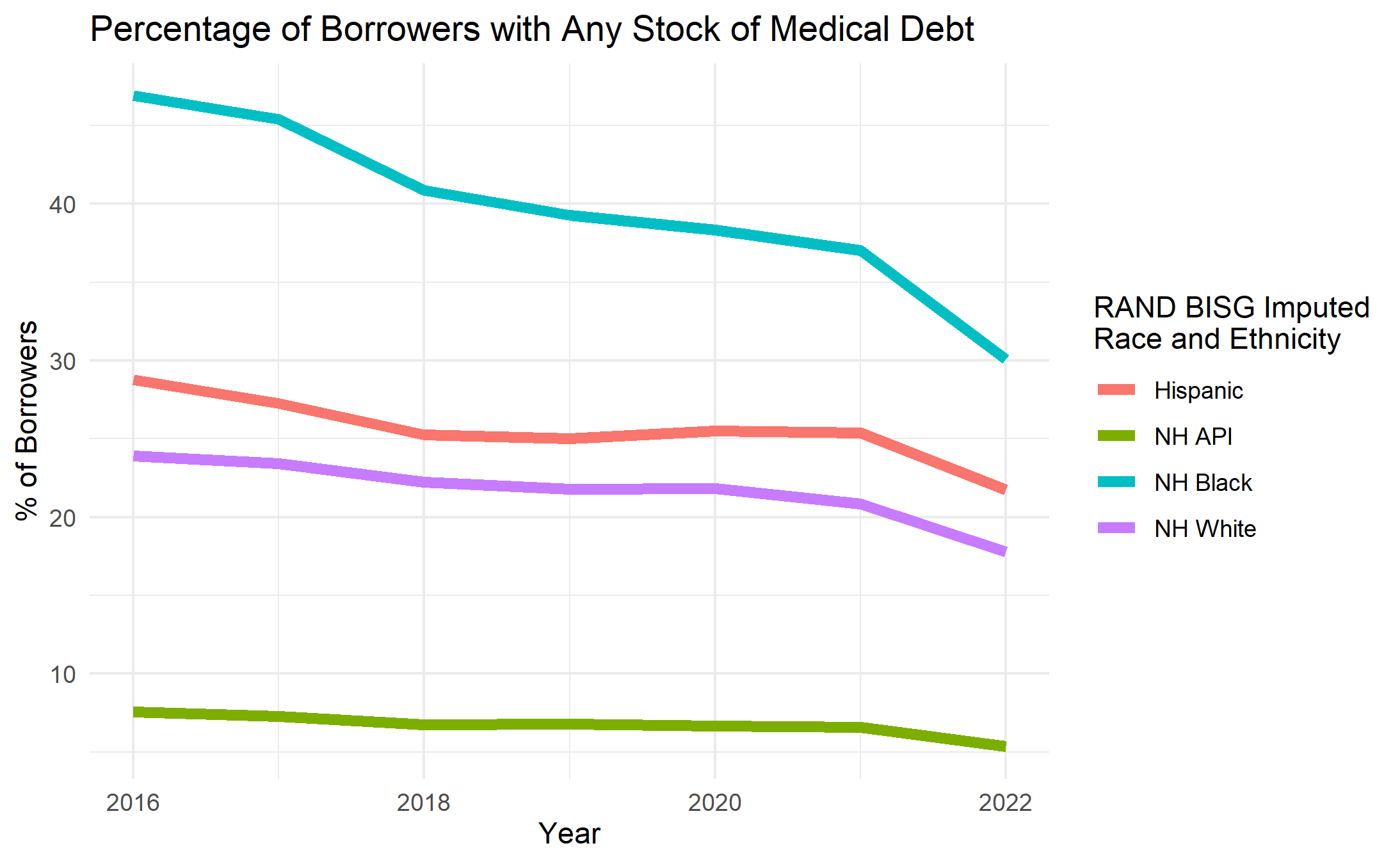


## Appendix A7. Trends in Predicted Probabilities/Amounts for Each Racial and Ethnic Group by State Medicaid Expansion Status

### Percent of borrowers with any annual flow


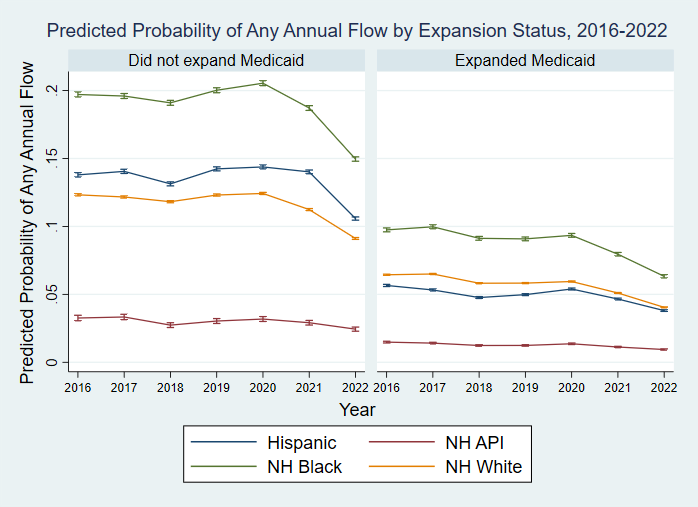


### Mean non-zero annual flow


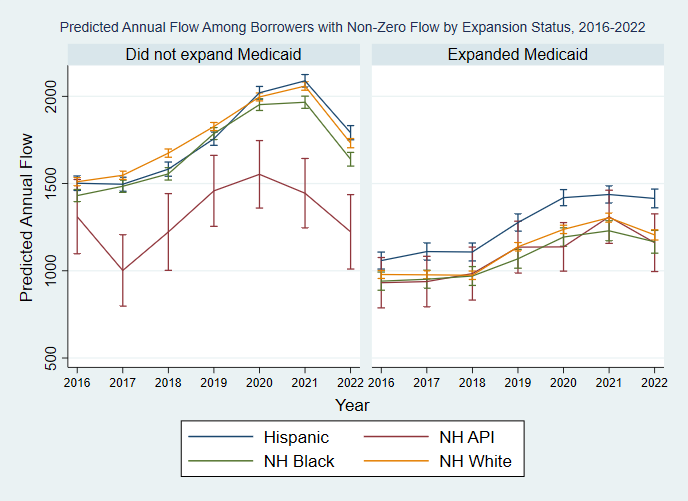


### Mean annual flow (all borrowers)


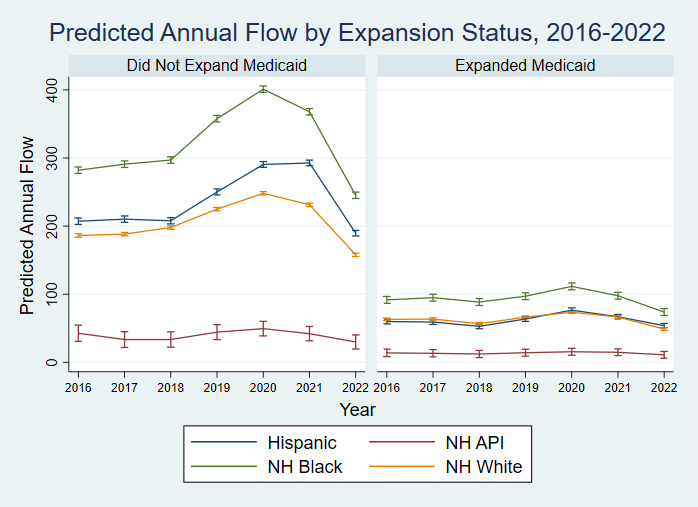


### Percent of borrowers with any stock


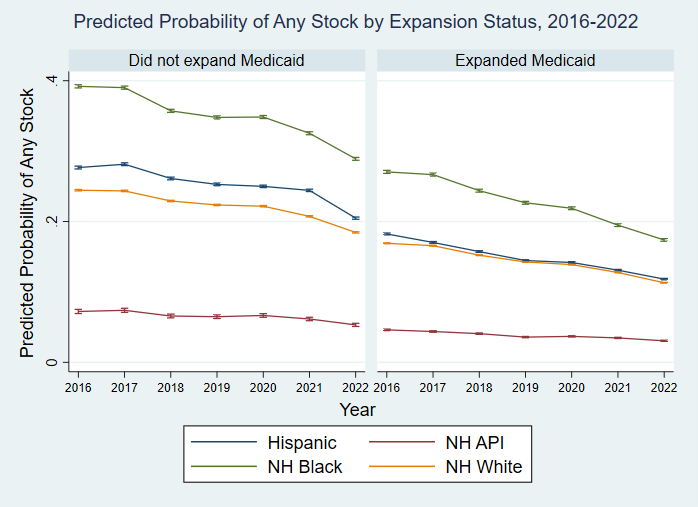


### Mean non-zero stock


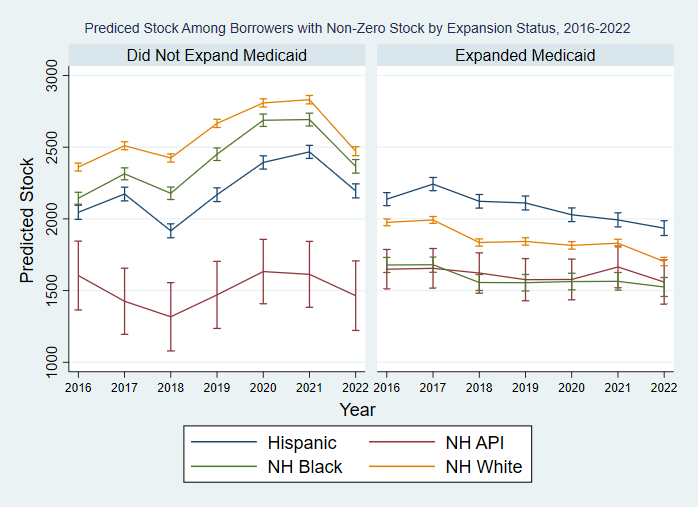


### Mean stock (all borrowers)


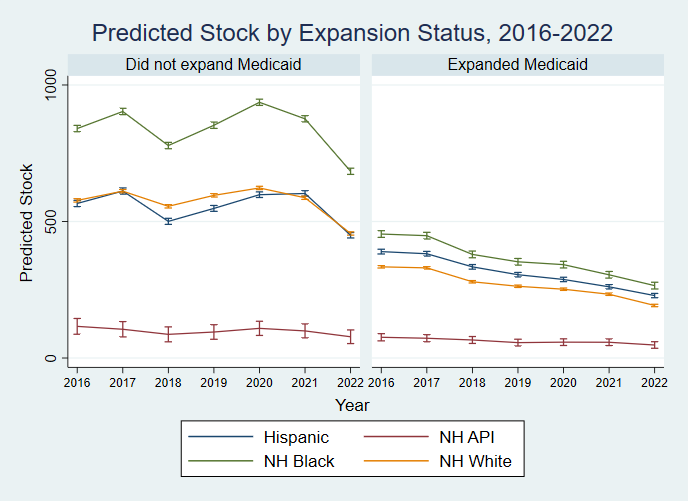


## Appendix A8. Comparing Single Classification vs Weighted Approaches

### Mean annual flow (all borrowers)


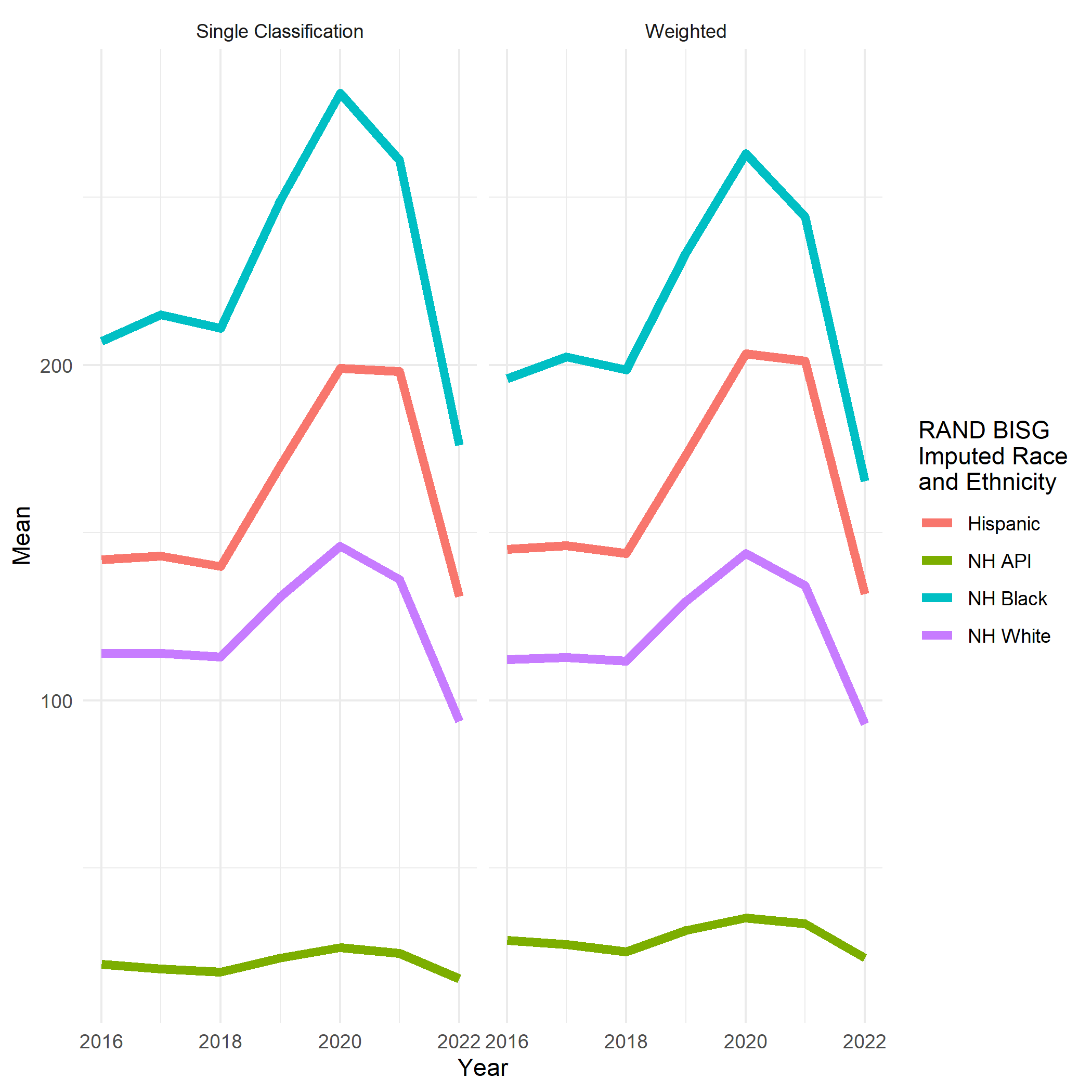


### Mean non-zero annual flow


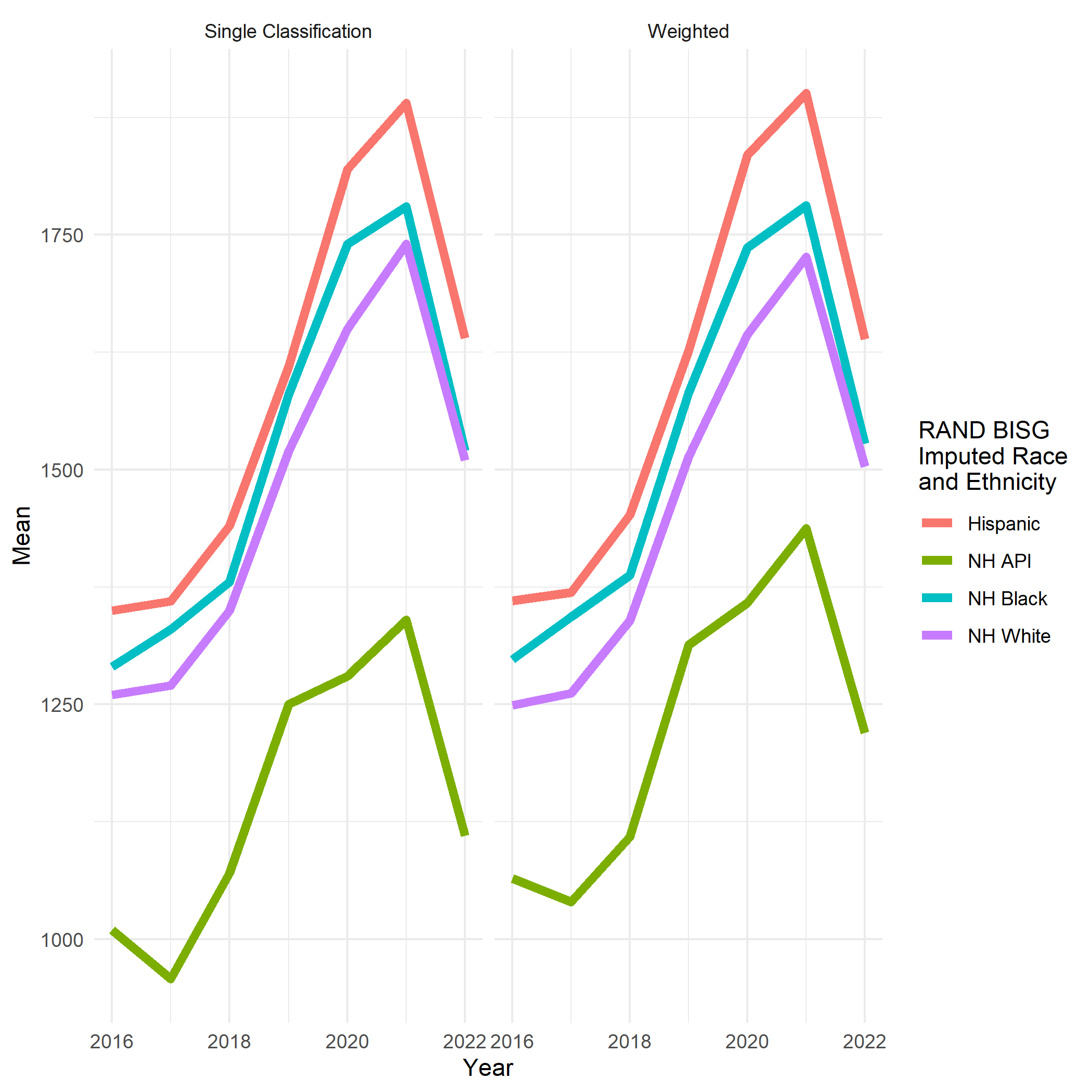


### Percent of borrowers with any annual flow


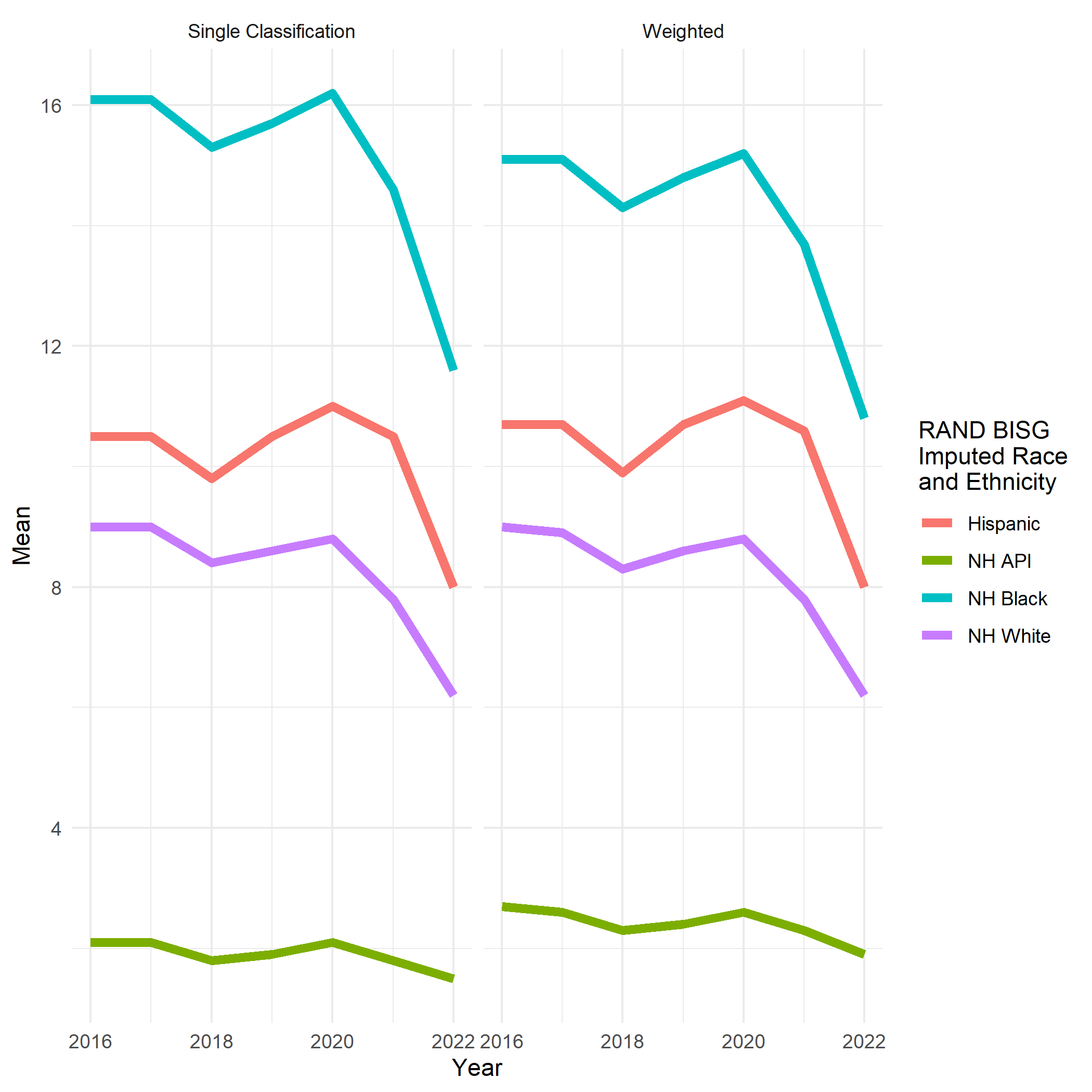


### Mean stock (all borrowers)


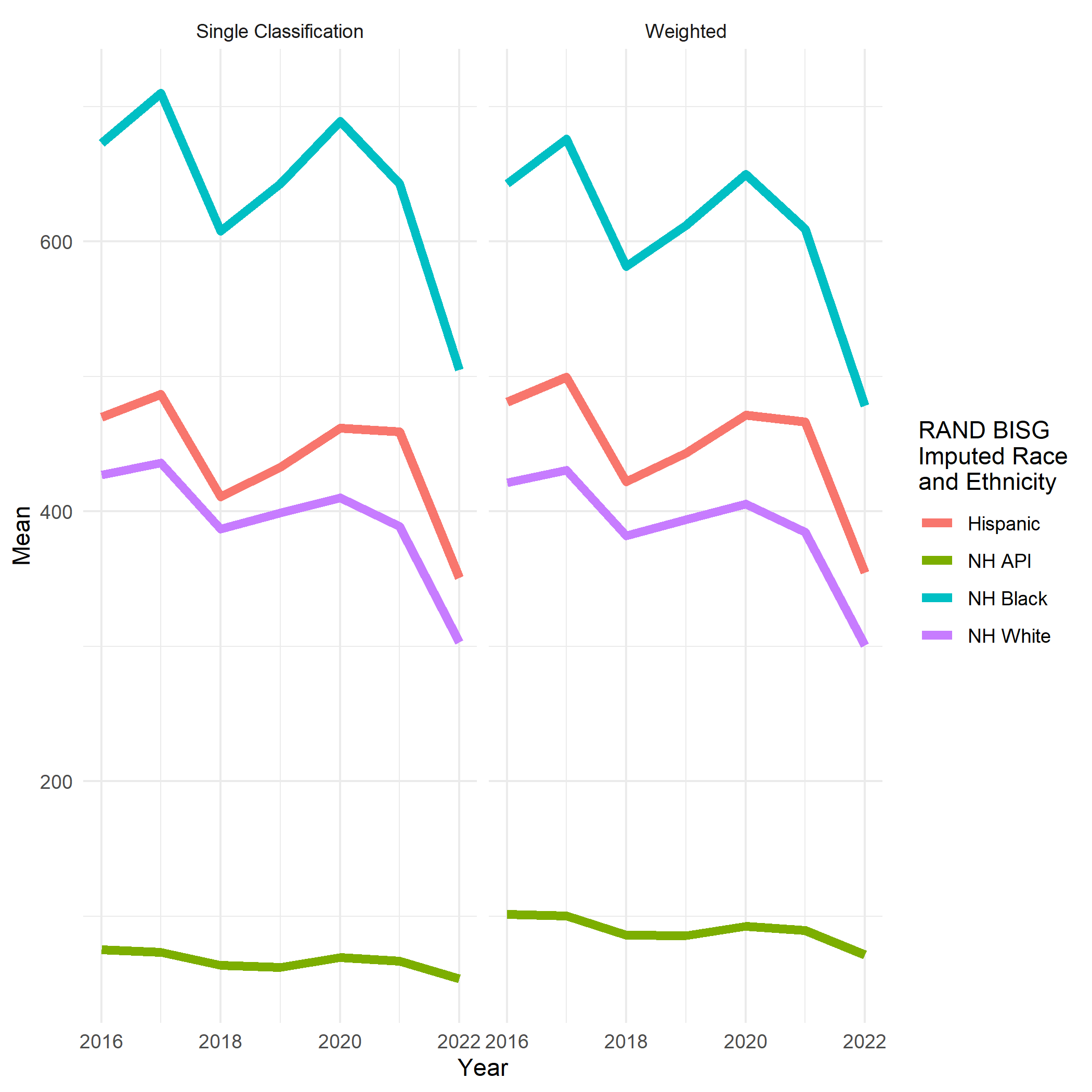


### Mean non-zero stock


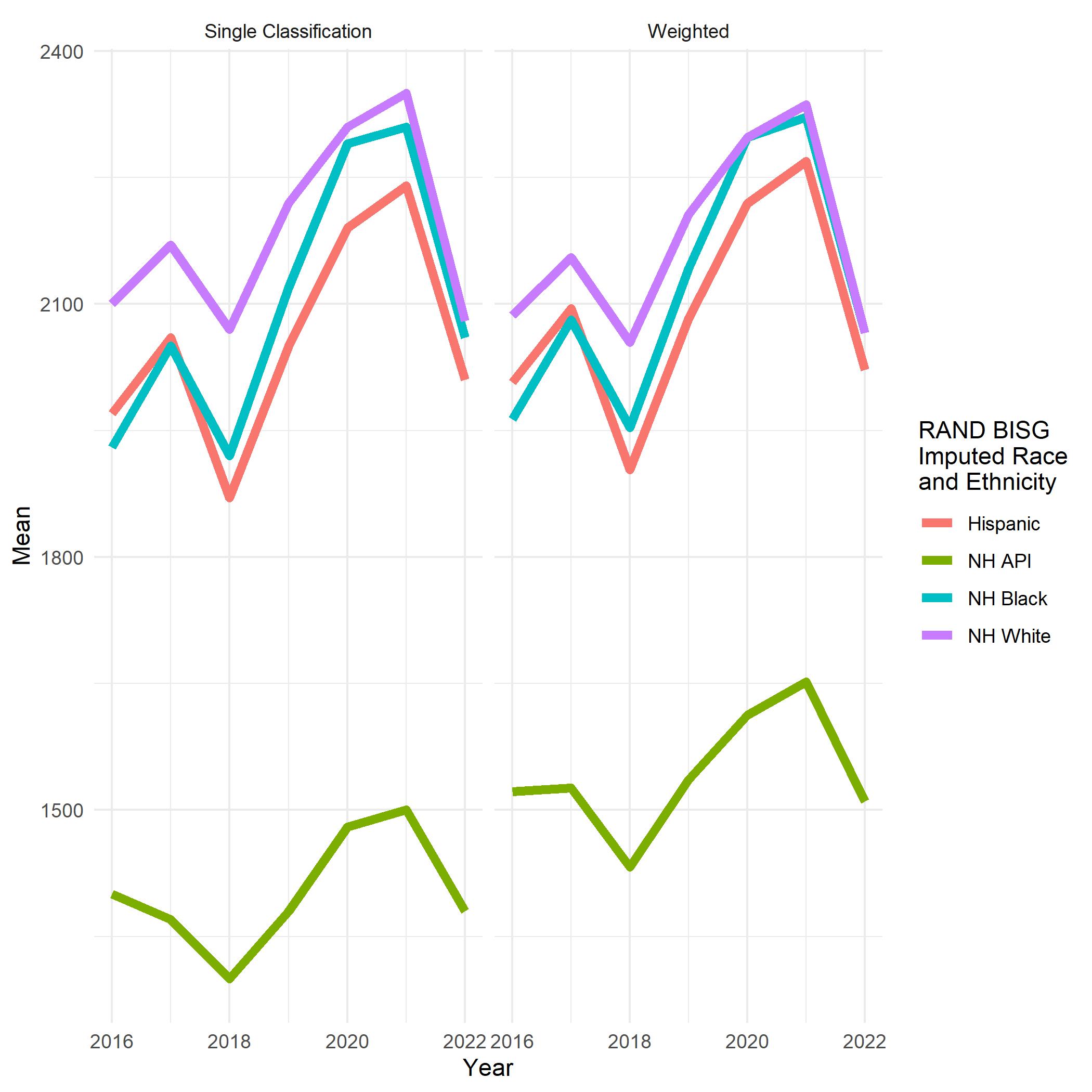


### Percent of borrowers with any stock


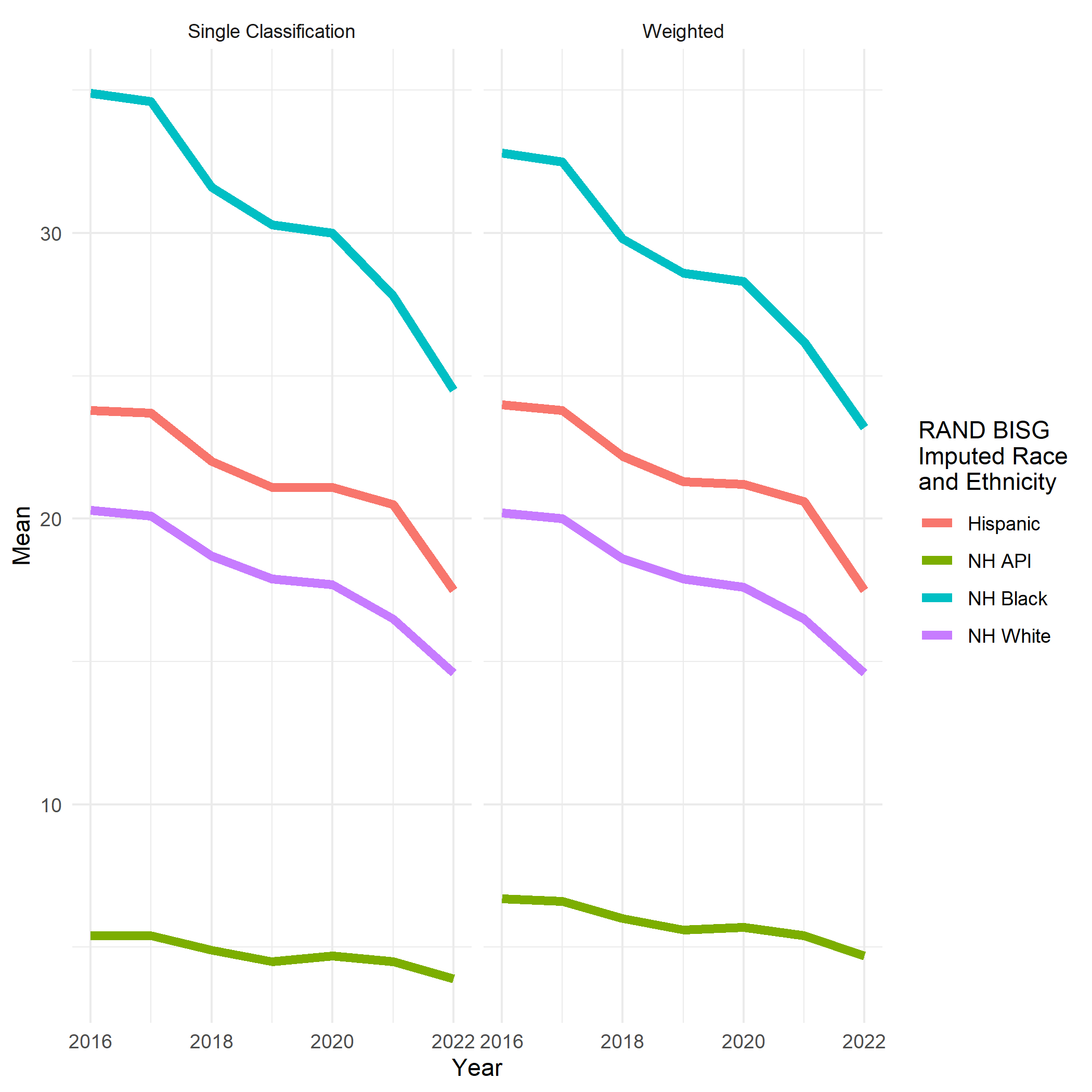


## Appendix A9. Assessing Differences in Debt by Missingness of RAND BISG Imputed Race and Ethnicity, 2016-2022


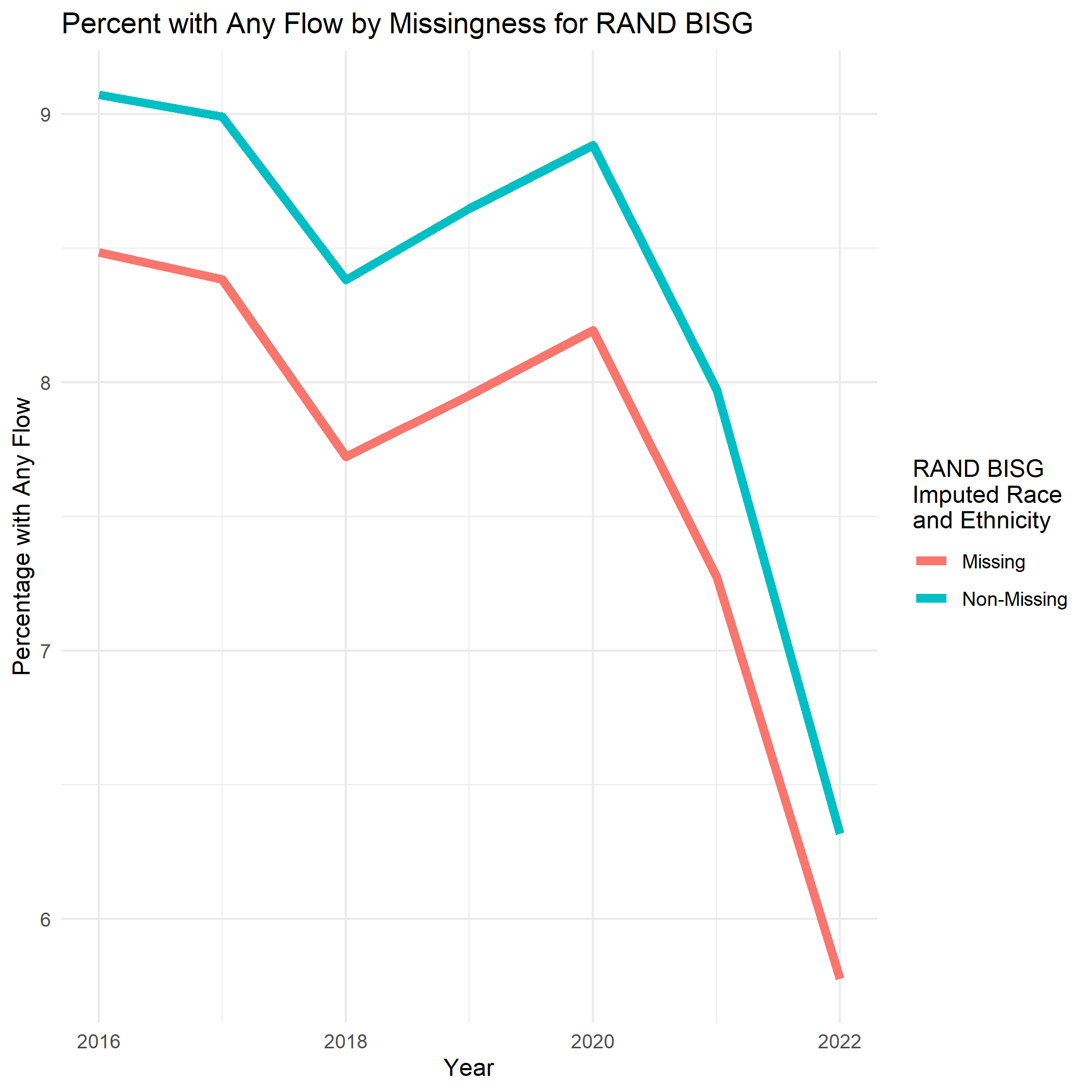


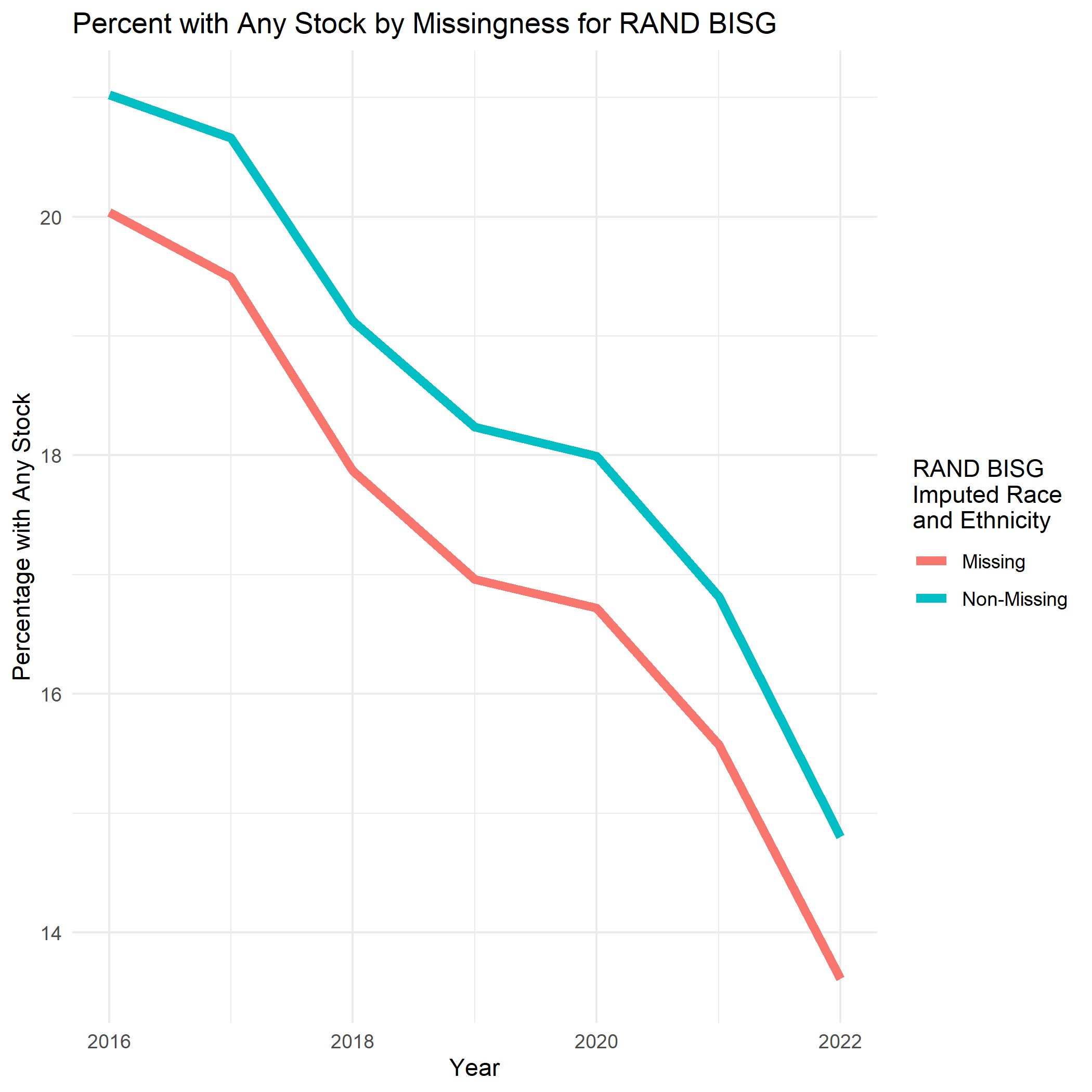


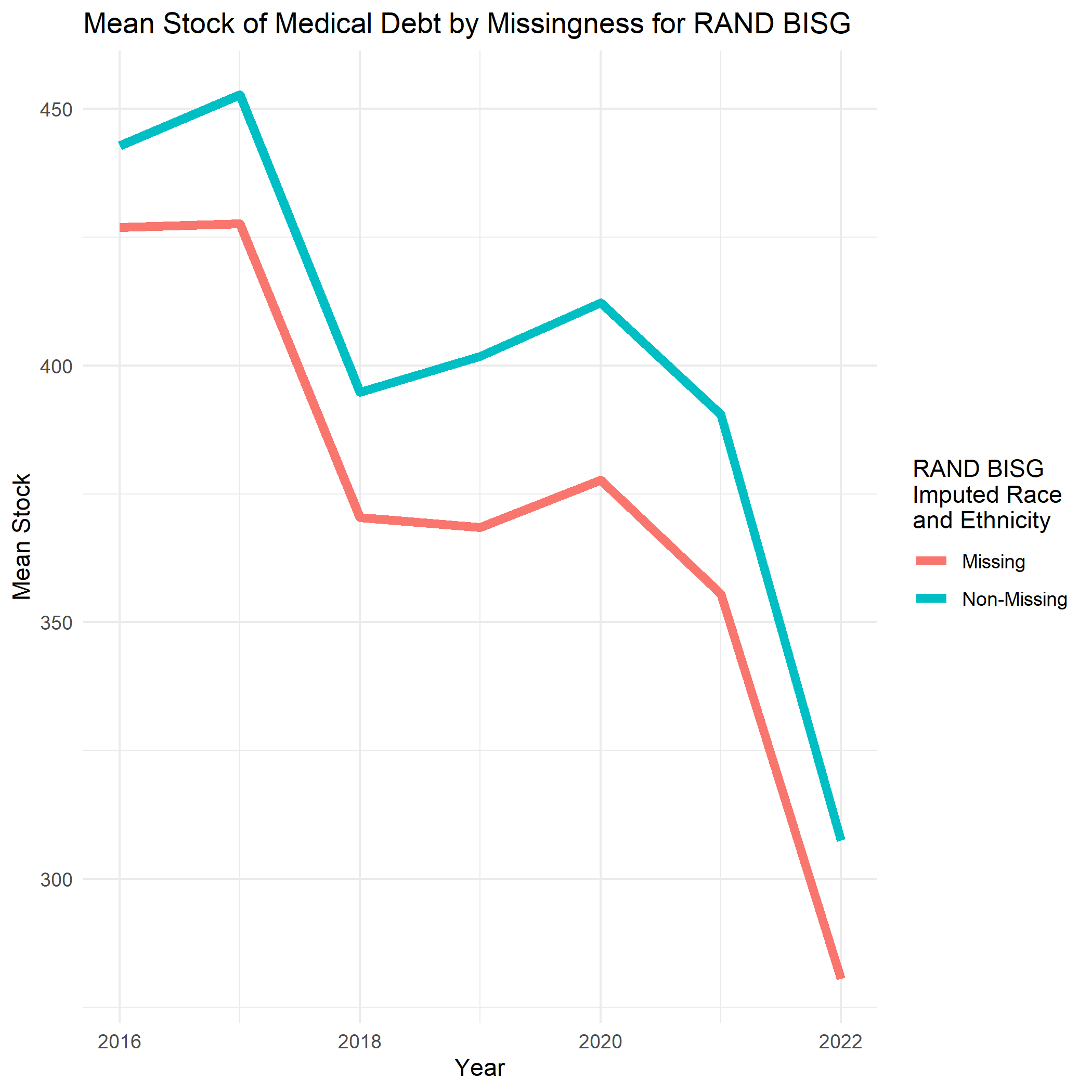


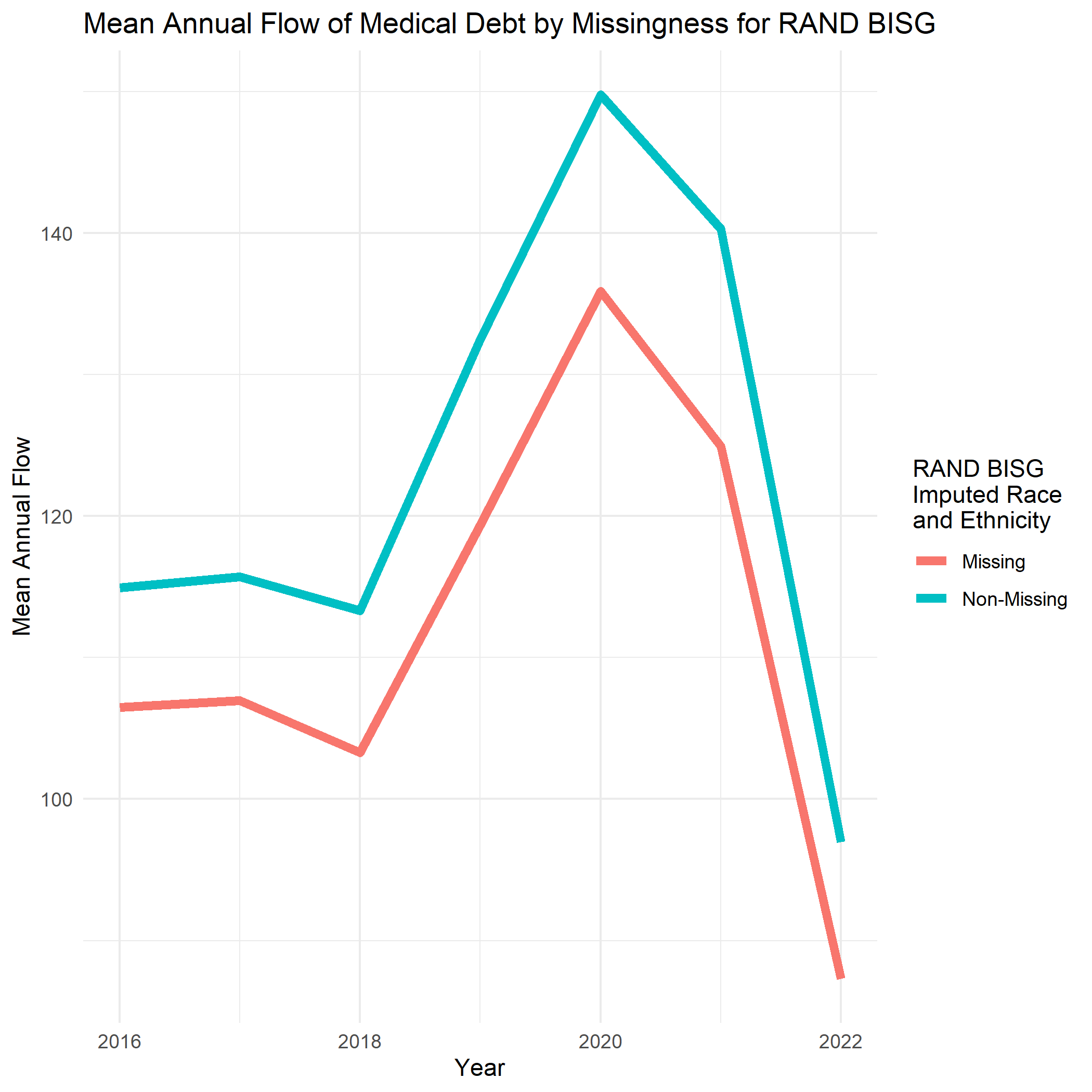


## Appendix A10. Differences in Medical Debt Across Imputed Race and Ethnicity Stratified by Income Decile

### Annual flow


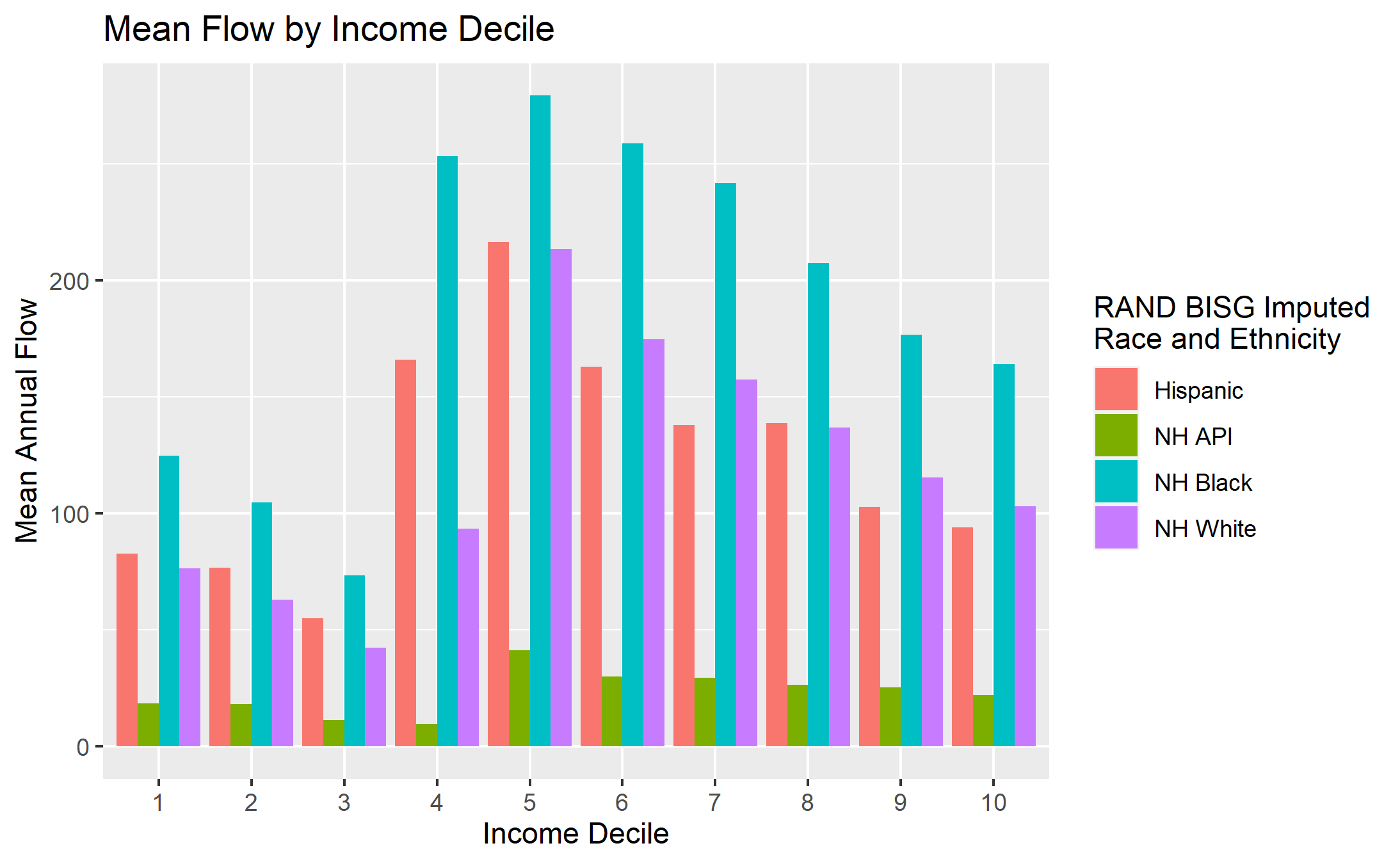


#### Non-zero flow


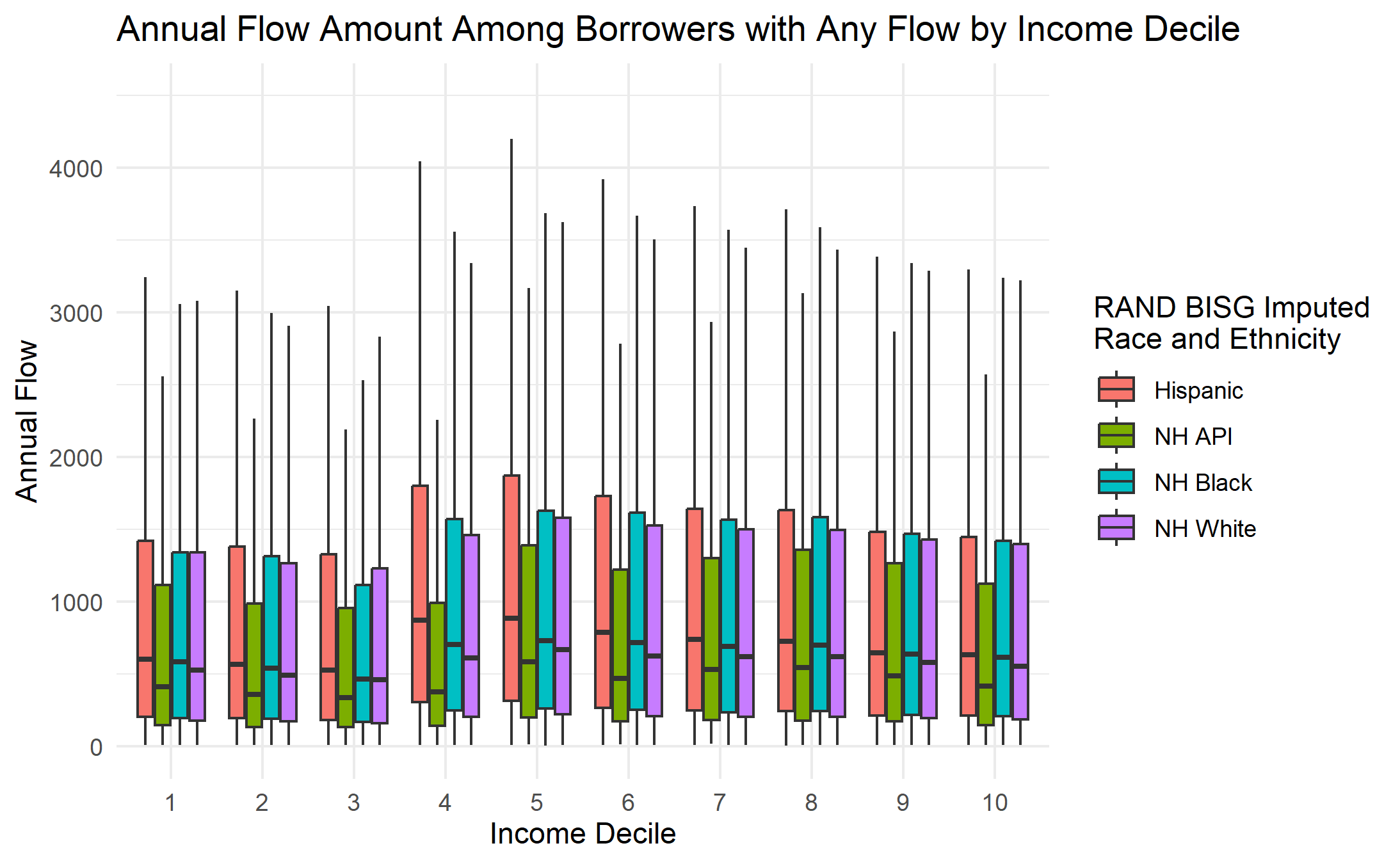


#### Percentage of borrowers with any Flow


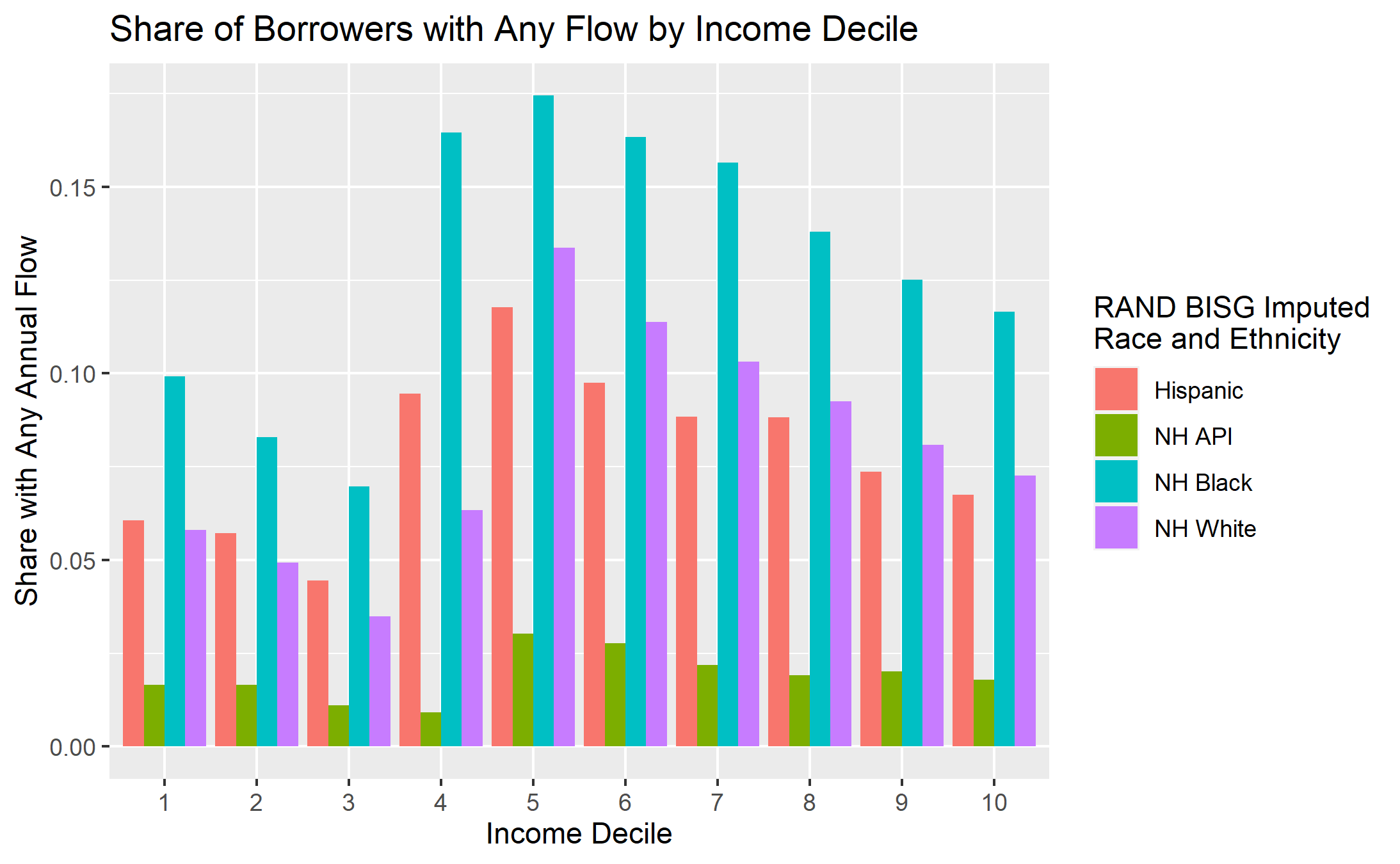


### Stock


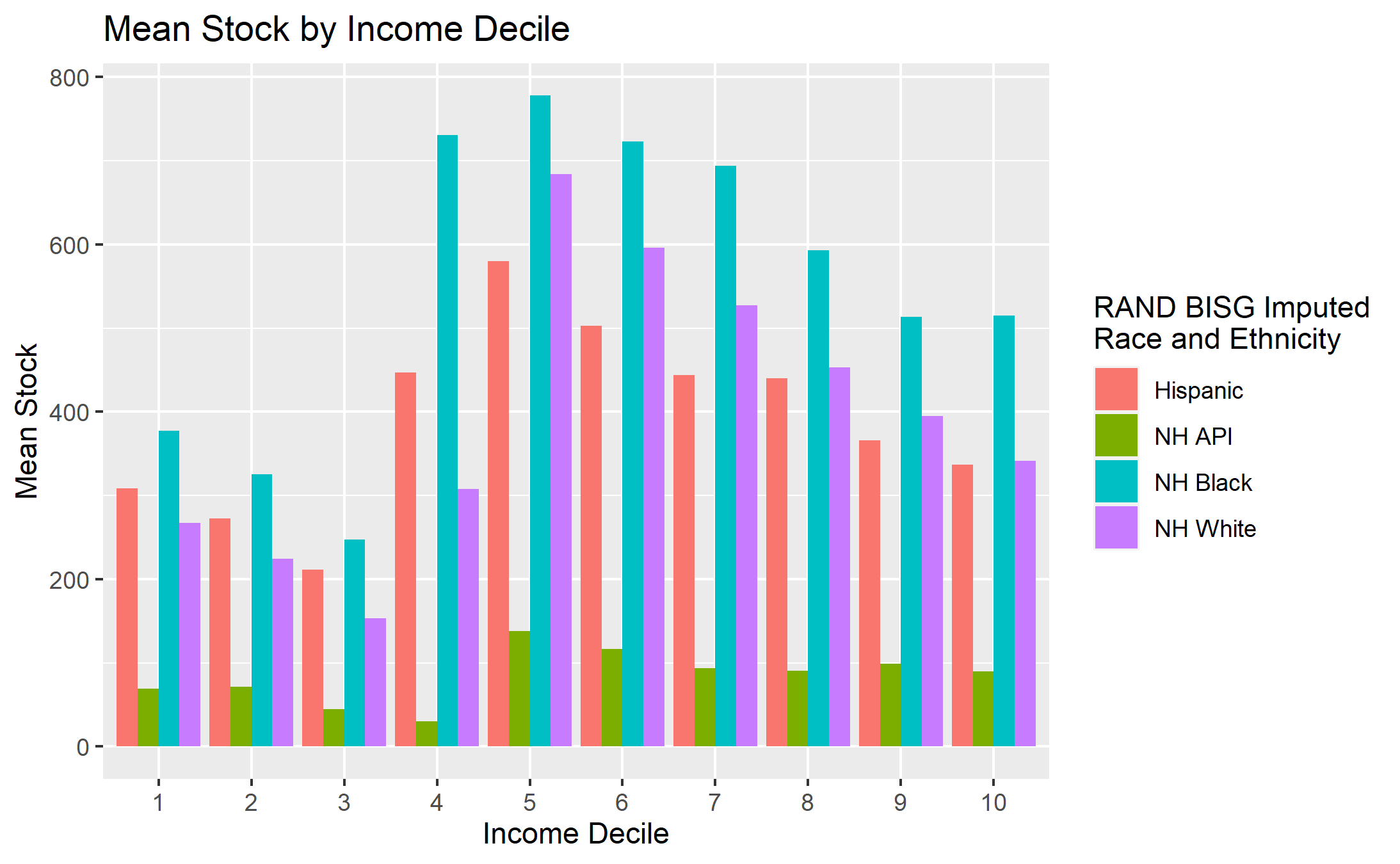


#### Non-zero stock


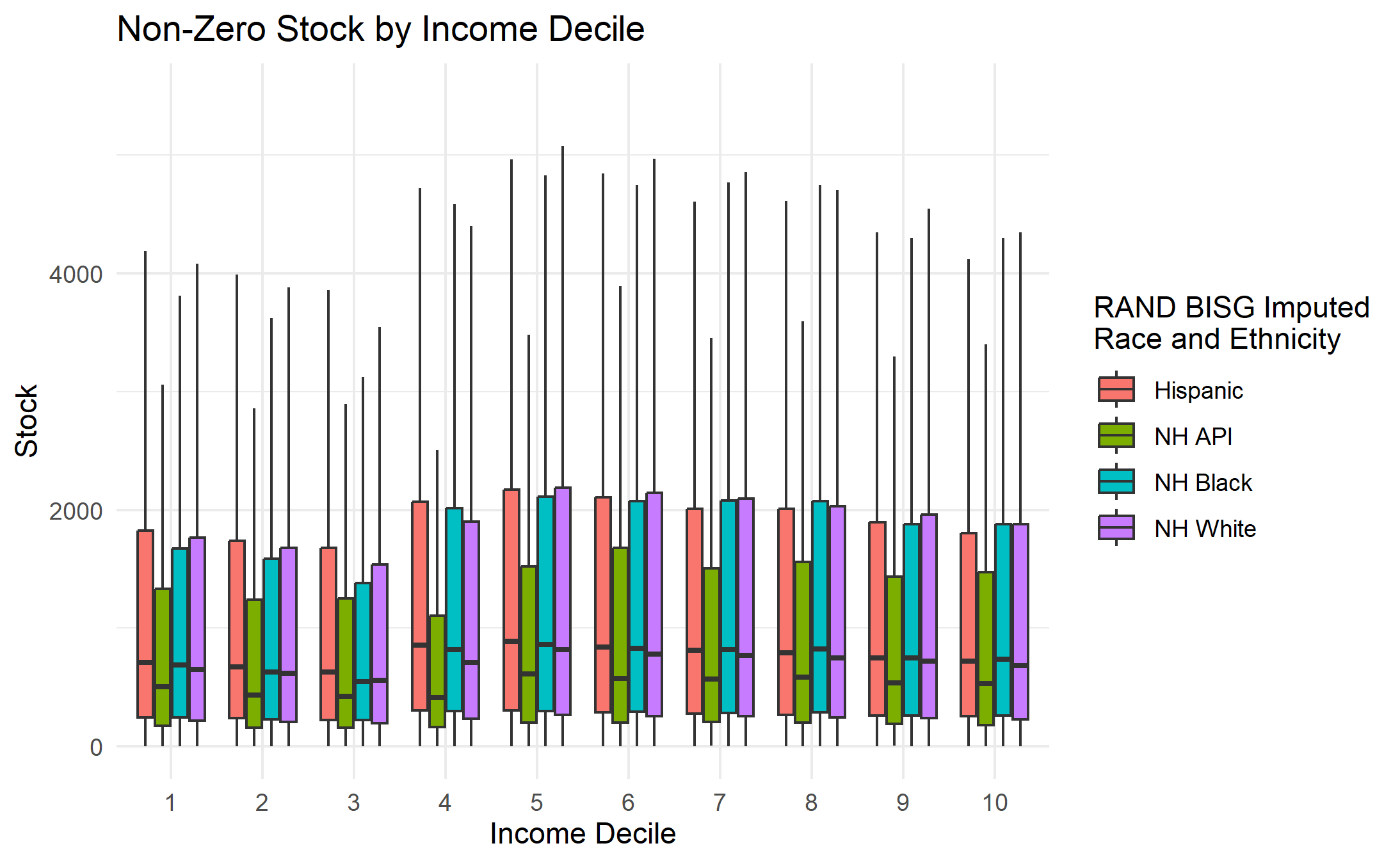


#### Percentage of borrowers with any stock


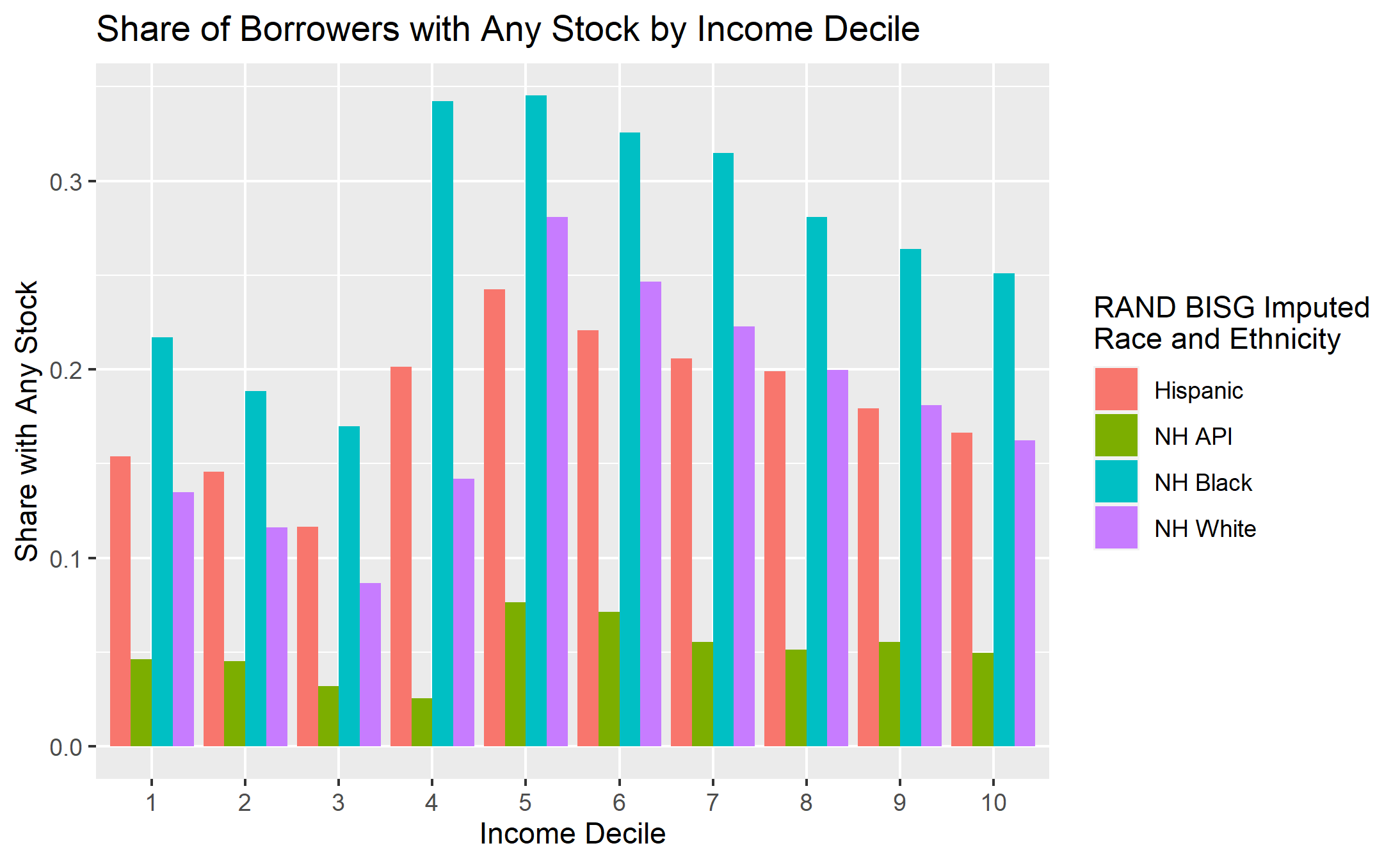


## 
